# Structural Aspects of Lithium‐Ion Conduction in the Phosphidotitanate Li_8_TiP_4_ and Its Comparison With Li_7+5_ *_x_* Ta_1−_ *_x_* P_4_ and Li_8−_ *_x_* Ti_1−_ *_x_* Ta*_x_* P_4_

**DOI:** 10.1002/chem.202503124

**Published:** 2026-01-22

**Authors:** David Müller, Tobias Kutsch, Sabine Zeitz, Viktor Hlukhyy, Gabriele Raudaschl‐Sieber, Wilhelm Klein, Thomas F. Fässler

**Affiliations:** ^1^ TUM School of Natural Sciences Chair of Inorganic Chemistry With Focus on New Materials Technical University of Munich Garching Germany; ^2^ TUM School of Natural Sciences Chair of Technical Electrochemistry Technical University of Munich Garching Germany; ^3^ TUMint·Energy Research GmbH Garching Germany; ^4^ TUM School of Natural Sciences Technical University of Munich, Chair of Inorganic and Metal‐Organic Chemistry Garching Germany; ^5^ TUM Catalysis Research Center Technical University of Munich Garching Germany

**Keywords:** all‐solid‐state battery, ion conductor, lithium, phosphorous, titanium

## Abstract

The chemical system Li/Ti/P has previously been subject to intensive investigation. However, reliable structural data for the reported phases have remained elusive. Motivated by the growing interest in phosphorus‐based lithium‐ion conductors, we have reinvestigated the synthesis, crystal structure, and physical properties of Li_8_TiP_4_. Phase pure Li_8_TiP_4_ was obtained, which crystallizes in the tetragonal space group *P*4_2_ *mc* (no. 105) with *a* = 8.37581(2) Å and *c* = 5.90489(2) Å. According to a structure determination from X‐ray diffraction powder data, Li_8_TiP_4_ is closely related to known Li_8_ *Tt*P_4_ with *Tt* = Si, Ge, and Sn, but adapts a different structure type. Basic structural findings are confirmed by solid state ^6^Li and ^31^P NMR spectroscopy. DFT calculations reveal a band gap of 2.5 eV and a good correlation between theoretical and experimental Raman spectra. From potentiostatic impedance spectroscopy an ion conductivity of (4.3 ± 0.6) × 10^−6^ S∙cm^−1^ at 298 K was found. In addition to the investigation of ternary Li_8_TiP_4_, an isotypic quaternary Ta‐containing phase is observed and studied by single crystal structure determination. Special emphasis in this study is placed on the role of Li occupancy in the voids of the cubic close‐packed (ccp) P atom arrangement and its impact on the ionic conductivity, in comparison to the known compounds Li_7+5_ *_x_* Ta*_x_* P_4_ and Li_8−_ *_x_* Ti_1−_ *_x_* Ta*_x_* P_4_ and traced back to the difference in crystal symmetry. Possible diffusion pathways of the Li^+^ ions were approached by BVSE calculations.

## 1 Introduction

All solid‐state batteries are heavily discussed in the search for a new generation of safe and powerful mobile energy storage devices. Replacing the flammable organic electrolyte in conventional lithium‐ion batteries with a solid‐state ion conductor is expected to provide several key advantages. First, the exclusive mobility of cations increases the lithium‐ion transference number and should enable fast charging without the buildup of diffusion potentials. Second, solid electrolyte layers can act as mechanical barriers against dendrite penetration, allowing the safe use of lithium‐metal anodes and thereby improving overall battery safety and performance. With all their proclaimed benefits, new technologies bring new challenges. In a true all‐solid‐state battery solid‐solid contacts predominate, interfaces and interphases play the most important roles and provision of electrical as well as ionic contact to all relevant components is a major objective. While the research interest increases, many lithium containing materials are being reviewed for their possible use as battery materials.

The solid electrolyte is a key component, which can either be a crystalline solid, an amorphous glass, an ion conducting polymer or a ceramic/polymer composite. Concentrating on crystalline solid materials, there are several compound classes, which promise to approach the numerous requirements in terms of safety, costs, electrochemical performance and stability from different sides. While garnet‐type electrolytes such as Li_7_La_3_Zr_2_O_12_ (LLZO) offer a wide electrochemical stability window and score in terms of safety, they exhibit only moderate ionic conductivity and are difficult to process at scale. Argyrodites such as Li_6_PS_5_Cl or Li_6_PS_5_Br are rather soft and conduct quite well, but are quite air and moisture sensitive. Thio‐LISICON for example Li_10_GeP_2_S_12_ (LGPS) still provide the highest ionic conductivities, but suffer of air and moisture sensitivity and are electrochemically stable only within a narrow potential window. NASICON and LISICON‐type conductors do not rank among the highest‐conductivity materials, but are rather stable and therefore safer as well. In general, oxide‐based conductors are more stable than sulfide‐based ones, but less conductive. So, beside its benefits, each material has its own drawbacks and the search for optimized or completely new material is far from an end [1, 2, 3].

Recently, reinvestigating the lithium phosphidotetrelates reported in the 1950ies [4] brought a new class of lithium ion conductors to light [5]. Generally, by replacing sulfide or oxide anions by phosphide, the charge of the anionic structure part is increased significantly, so these materials possess a much higher lithium content, which, provided that these lithium ions are mobile, results in a high charge carrier concentration. Several of the newly found compounds containing the tetrel (*Tt*) elements Si, Ge, and Sn can be expressed with the formula Li_36−4_ *_n_Tt_n_* P_12_ with *n* = (2, 3, 4, 5, 6, 8). As a common feature of all these compounds, their crystal structures comprise a cubic close packing of P atoms and contain the tetrahedral [*Tt*P_4_]^8−^ unit as major building motive. With increasing *n* linked tetrahedral units occur which can be interpreted as a condensation of these tetrahedra formally eliminating Li_3_P. In the Li_14_ *Tt*P_6_ compounds [6, 7], beside the tetrahedral units isolated P^3−^ units exist as well, in Li_8_ *Tt*P_4_ the [*Tt*P_4_]^8−^ tetrahedra are isolated within the structure and seize ordered positions in two modifications (named as α‐ and β‐phase) [5, 8, 9], in Li_10_ *Tt*_2_P_6_ two tetrahedra form edge‐sharing dimeric units [10], in Li_2_ *Tt*P_2_ the tetrahedra build two interpenetrating nets of connected supertetrahedra of four vertex‐sharing *Tt*P_4_ units [5, 11, 12], and in Li*Tt*_2_P_3_ even larger supertetrahedra are formed [11]. Whilst this chemistry is most interesting from a structural point of view, the electrochemical properties in the lithium rich region with the structures containing isolated tetrahedral units are most relevant. The high symmetric Li_14_ *Tt*P_6_ phase shows ionic conductivities up to 1.7 mScm^−1^ [6, 7]. In the composition Li_8_ *Tt*P_4_ there exist two modifications, arising from two different ways of ordering the isolated [*Tt*P_4_]^8−^ tetrahedra. While Li_8_SiP_4_ only forms the alpha phase, Li_8_GeP_4_ and Li_8_SnP_4_ are accessible in both modifications, with the beta modification as a metastable high‐temperature phase. The beta phase shows higher ionic conductivity, reaching 0.66 mScm^−1^ for Li_8_SnP_4_ [5, 8, 9].

Expanding the structural variety and electrochemical properties of this system, we started taking also transition metal into consideration [13]. As the titanium group is chemically related to the tetrel group, we were interested in the structure and electrochemical properties of compounds in which titanium replaces Group‐14 elements. There exist several investigations on compounds in the Li‐Ti‐P system which also lead to controversial discussions: In the 1950s *Juza* reported Li_5_TiP_3_, adopting the antifluorite structure type with P atoms fo ming a cubic close‐packing (*ccp*) with Li and Ti atoms are disordered at tetrahedral voids, together with a variety of lithium phosphidotetrelates and lithium arsenidotetrelates [4]. Later in 1968 also the lithium phosphidotitanates Li_9_TiP_4_ and Li_8_TiP_4_ were reported [14]. While Li_9_TiP_4_ represents a disordered antifluorite type structure, Li_8_TiP_4_ is merely described as a tetragonally distorted, pseudo cubic 2×2×2 superstructure of the antifluorite type without further structural details provided [14]. In the early 1990s *Schuster* reinvestigated the disordered antifluorite type Li_9_TiP_4_ and described this compound with slightly deviating stoichiometry as Li_11_TiP_5_ [15]. While *Juza* claims a mixture of Ti^2+^ and Ti^4+^ resulting in the overall oxidation stage +3, in the formula of *Schuster* only Ti^4+^ occurs, which seems more likely. The measured lattice parameters differ by just 0.01 Å (*Juza*: 5.96 Å, *Schuster*: 5.97 Å), so it seems probable that the same phase has been described in both papers. Comparing both structures to the corresponding tetrelates, the lithium content in the octahedral voids is lower than in the Li_14_ *Tt*P_6_ phases. Chemically and structurally related to lithium phosphidotitanate, the alkaline earth phosphidotitanates Ba_4_TiP_4_ and Sr_4_TiP_4_ are isostructural to the alkaline earth phosphidotetrelates *Ae*_4_ *Tt*P_4_ (*Ae* = Ca, Sr, Ba; *Tt* = Si, Ge, Sn) which show the same 2×2×2 superstructure of the antifluorite type unit cell with the tetrahedral ordering of the beta Li_8_ *Tt*P_4_ phases (*Tt* = Ge, Sn) but with all the alkaline earth ions occupying octahedral voids [16, 17].

In the early 2000s, lithium phosphido transition metallates were investigated as anode materials for lithium ion batteries. Li*_x_* TiP_4_ can reversibly incorporate five lithium equivalents with *x* = 3 to 11, which results in a theoretical capacity of 830 mAhg^−1^. The investigated phase width confirms the structure of Li_9_TiP_4_ which is regarded as a disordered high temperature phase. During discharging, the material loses its crystallinity, reforming reversibly during charging. The redox reaction is proposed to take place at the P‐Ti molecular orbital rather than just on the transition metal, accounting for the extreme formal oxidation states as anticipated for this process [18, 19, 20, 21, 22].

Here we report on the reinvestigation of the synthesis as well as the spectroscopic and structural characterization of pristine Li_8_TiP_4_. Special emphasis is placed on the role of Li occupancy in the voids of the ccp P atom arrangement and its impact on the ionic conductivity, in comparison to the known compounds Li_7_TaP_4_ and Li_9.5_Ta_0.5_P_4_. Possible diffusion pathways of Li^+^ ions are analysed using BVSE calculations. Additionally, we report an isotypic quaternary compound Li_8−_ *_x_* Ti_1−_ *_x_* Ta*_x_* P_4_.

## 2 Experimental

### 2.1 General

All steps of synthesis and sample preparation were performed inside an argon‐filled glove box (MBraun, *p*(H_2_O), *p*(O_2_) < 1.2 ppm) or in containers sealed under Ar atmosphere. Reaction vessels have been either sealed under argon or evacuated.

### 2.2 Synthesis

Microcrystalline powder of phase‐pure Li_8_TiP_4_ was synthesized in a two‐step synthesis via ball milling with subsequent heat treatment. Lithium (Rockwood, 99 %), titanium powder (99 %) and red phosphorus (98 %) were weighed in stoichiometric ratio into a tungsten carbide milling jar (45 mL, Fritsch) with six tungsten carbide balls (Ø = 1.5 cm) and milled for 18 h at 350 rpm. The received powder was pressed into pellets, sealed under vacuum in a graphitized quartz ampoule and heated for 24 h to 973 K. Finally, the substance is obtained as a grey brittle melt cake crumbling under light pressure into small crystallites which show red color at closer view under a microscope.

Another method of synthesis was applied aiming at the growth of larger single crystals, but this inadvertently resulted in samples contaminated with tantalum. Lithium, titanium and excess of phosphorus were weighed into a tantalum crucible, seal‐welded under argon, heated to 973 K for 24 h and cooled with a rate of 0.1 Kmin^−1^ slowly to room temperature. The crystals appeared black with a dark red tinge. Due to the excess of phosphorous, TaP was obtained as a byproduct.

### 2.3 Powder X‐ray Diffraction Data

Li_8_TiP_4_ was characterized by powder X‐ray diffraction (PXRD) on a STOE Stadi‐P with Cu *K_α_*_1_ source (*λ* = 1.54058 Å), curved Ge(111)‐monochromator and DECTRIS MYTHEN 1K detector. Samples were sealed under argon in a 0.3 mm glass capillary and measured in Debye‐Scherrer geometry. Ab initio structure solution from powder diffraction was performed with the program Superflip, as implemented in Jana2020 [23]. While different space groups were tried, only very few were possible to refine including *P*4_2_/*nmc* as the highest symmetric one, which was chosen therefore. Rietveld analysis has been done with the Fullprof Suite [24]. Peak profile shape was described by a pseudo‐Voigt function; the background of the diffraction pattern was fitted using a linear interpolation between selected data points in nonoverlapping regions. Scale factor, lattice parameters, fractional coordinates of atomic sites and their isotropic displacement parameters, profile shape parameters, and half width (Caglioti) parameters were varied during the Rietveld refinement. Crystallographic details and refined parameters are given in Tables 1, 2. In space group *P*4_2_ *mc*, no symmetry restrictions are existent for the atom positions relative to the *c* axis, so the structure keeps wandering along *c* during the refinement. Therefore, the *z* parameter of the titanium atom was restrained to a fix value and, thus, this parameter has no standard deviation. Crystallographic data for the structure of Li_8_TiP_4_ has been deposited with the Cambridge Crystallographic Data Centre, CCDC, 12 Union Road, Cambridge CB21EZ, UK (Fax: +44‐1223‐336‐033; e‐mail: deposit@ccdc.cam.ac.uk, http://www.ccdc.cam.ac.uk) and can be obtained free of charge on quoting the depository number CSD‐2465967.

**TABLE 1:** Details of the Rietveld structure refinement of Li_8_TiP_4_ on X‐ray powder data.

formula	Li_8_TiP_4_
formula weight	227.30 g mol^−1^
temperature	293 K
crystal system	tetragonal
space group	*P*4_2_ *mc* (no. 105)
unit cell	*a*,*b* = 8.37581(2) Å
	*c* = 5.90489(2) Å
*Z*	2
*V*	414.252(2) Å^3^
*ρ*_calc_/	1.822 g cm^−3^
*λ*	Cu *K_α_*_1_ (1.540598 Å)
*μ*	15.192
2*θ* range	5°–90°
*R_p_*	3.37
*R_wp_*	4.82
R_exp_	3.07
*χ* [2]	2.460
*R_Bragg_*	2.30
*R_f_*	2.36
depository no.	CSD‐2465967

**TABLE 2:** Atomic coordinates, isotropic displacement parameters, and site occupation factors for Li_8_TiP_4_ from Rietveld refinement.

Atom	Wyckoff position	*x*	*y*	*z*	*U_iso_*	s.o.f.
Ti	2*c*	0	1/2	0.25	0.0066(5)	1
P1	4*e*	0.2311(4)	1/2	0.0143(16)	0.0077(15)	1
P2	4*d*	0	0.2580(4)	0.4657(18)	0.0059(13)	1
Li1	2*c*	0	1/2	0.723(7)	0.008(5)	1
Li2	8*f*	0.2687(12)	0.2500(12)	0.237(5)	0.008(3)	1
Li3	2*b*	1/2	1/2	0.284(4)	0.021(6)	1
Li4	4*d*	0	0.1751(16)	0.009(3)	0.053(5)	1

### 2.4 Single Crystal Structure Determination of Li_7.9_Ti_0.9_Ta_0.1_P_4_

Single crystals were isolated and sealed inside a glass capillary (Ø = 0.3 mm). Diffraction data were collected at room temperature, at 253 K, and at 150 K on a STOE StadiVari diffractometer (Mo *K_α_* radiation) equipped with a DECTRIS PILATUS 300K detector. The space group determination confirmed the results from powder data, the reflection conditions with *l* = 2*n* for 00*l* and *hhl* lead to the extinction symbol *P*(4_2_)‐–*c*. Considering the highest possible space groups according this condition, in *P*4_2_ *mc* the structure solution was successful without absence violations while in the also possible space groups *P* $\bar{4}$2*c* and *P*4_2_/*mmc* no solution succeeded. A structure solution was possible in *P*4_2_/*nmc*, too, but here numerous absence violations for *hk*0, +*k* = 2*n* were observed and the residual values were significantly higher. The structure was solved by Direct Methods (SHELXS‐97), confirming the structural model as obtained from powder X‐ray data, and was refined by full‐matrix least squares calculations against *F* ^2^ (SHELXL‐2014) [25]. During the structure determination, the Ti atom position turned out to be overstaffed with a refined site occupation of about 120 %. According to former results obtained from preparations including Ta crucibles, a contamination with small amounts of tantalum, stemming from the synthesis container, was assumed. In the present compound, tantalum partly replaces the titanium in the structure and, thus, the transition metal atoms share one crystallographic site and were refined with the same positional and displacement parameters. A free refinement of the occupation factors at this stage resulted in 91 % for the Ti and 7 % for the Ta occupation. According to the most probable oxidation state of +5 for Ta, some additional positive charge with respect to replaced Ti^4+^ must be present. Confirming this, a reduced lithium content lower than 90 % was observed for the Li4 atom site while for Li1, Li2, and Li3 site occupations between 98 % and 101 % were refined. The latter atoms were thus fixed to full occupation, while the occupation of the Li4 atom was refined. Finally, the highest residual electron density peak located from the Fourier map in sufficient distance to other atoms was added as the Li5 atom and refined with a partial occupation slightly below 10%. In the last refinement cycles, the occupation factors of the mixed Ti/Ta site and of the two affected Li atom positions were restrained to obtain charge balance. Therefore, the estimated uncertainties are mainly determined by the heavy transition metal atoms but might be higher for the involved Li atoms. The structure was refined as an inversion twin with a Flack parameter close to 0.5 and the crystallographically determined composition resulted in Li_7.9363(6)_Ti_0.9363(6)_Ta_0.0637(6)_P_4_. All atoms were refined with anisotropic displacement parameters. In the refinements of the data recorded at r.t. and at 253 K, the displacement parameter of the minor occupied Li atom had to be restrained with an ISOR command. Same as in the structure determination from powder data, the *z* parameter of the Ti/Ta atom was fixed to avoid continuous move of the whole structure along the *c* axis during the refinement. The crystallographic data for the single crystal data are summarized in the supporting information. Further details of the crystal structure investigations may be obtained from the joint CCDC/FIZ Karlsruhe online deposition service via www.ccdc.cam.ac.uk/data_request/cif, on quoting the depository numbers CSD‐2465968 to CSD‐2465970.

### 2.5 Impedance Spectroscopy

The ionic conductivity of Li_8_TiP_4_ was determined by potentiostatic electrochemical impedance spectroscopy (PEIS) using an in‐house designed cell setup with ion blocking electrodes. Basically, the setup consists of a stainless‐steel casing, a PEEK tube as sample container, hardened stainless‐steel dies as current collectors and pistons comprising a gasket for sealing the cell as well as six screws for tightening the cell. To ensure the complete tightness of the setup, the pistons are additionally greased. As pressure, we applied an operation pressure of 450 MPa by fastening the screws (M14) with a defined torque of 30 Nm. For more detailed information on the cell design, please check references [5, 6].

To determine the ionic conductivity, powdered samples of Li_8_TiP_4_ (≈ 400 mg) were placed between the two 8 mm dies as ion blocking electrodes and compressed by the cell pressure to ≈ 88 % of their crystal density. The resulting thickness of the thus compressed samples was determined with a precision caliper by measuring the distance between both current collectors using six holes in each current collector, which are orientated in a symmetric configuration. For data collection a Bio‐Logic potentiostat (VMP‐300) in the frequency range between 7 MHz and 100 mHz and a potentiostatic excitation of ±10mV was used. The resulting impedance spectra were fitted using the RelaxIS 3 software (rhd instruments).

For determination of the electronic conductivity, chronoamperometry (CA) measurements were performed using the same two‐electrode‐cell setup by polarizing the sample with three potential steps of 50 mV, 100 mV, and 150 mV for 6 h while recording the current response. For data treatment the software OriginPro 2020b was used. PEIS and CA measurements were performed in a climate chamber (ESPEC, LU‐114). For the determination of the activation energy of the Li^+^ conduction as well as the electronic conductivity, the cell temperature was set to 273, 283, 298, 313, 328, 343, and 353 K. Prior to PEIS/CA measurements, the cell rested at the desired temperature for 180 min to allow for thermal and mechanical equilibration.

### 2.6 Solid‐State NMR Spectroscopy

Magic angle spinning (MAS) NMR spectra have been recorded on a Bruker Avance 300 NMR device operating at 7.04 T by the use of a 4 mm ZrO_2_ rotor. The resonance frequencies of the investigated nuclei are 44.167, and 121.495 MHz for ^6^Li, and ^31^P, respectively. The nutation frequency was set to 11 kHz (^31^P), 13 kHz (^31^P), and 15 kHz (^6^Li and ^31^P).

### 2.7 Raman Spectroscopy

Raman spectra were recorded on an inVia Raman microscope (Renishaw, RE04) with CCD detector, 50‐fold magnifying objective and a grating with 1800 lines mm^−1^. The powdered sample was sealed in a 0.3 mm glass capillary and excited with a 785 nm laser beam for 1 s at 0.1%  laser. For the final spectrum, 120 single measurements were averaged. The software WiRe 4.2 (build 5037, Renishaw 2002) was used for data recording [26]. The wavenumbers of the spectrum have been scaled by a factor of 0.94 to correct the typical overestimation of predicted harmonic frequencies [27]. For the calculation the crystallographic data were adjusted to contain no underoccupied lithium positions, the temperature and wavelength were set corresponding to the experimental values (*T* = 298.15 K, *λ* = 785 nm).

### 2.8 DFT Calculations

The computational analysis of Li_8_TiP_4_ was performed using the CRYSTAL17 program package and hybrid density functional methods [28, 29]. A hybrid exchange‐correlation functional after Perdew, Burke, and Ernzerhof (DFT‐PBE0) was used [30]. Localized, Gaussian type triple ζ‐valence + polarization level basis sets were used for Ti and P and split valence + polarization level basis sets for Li. The basis sets were derived from the molecular Karlsruhe basis sets [31, 32, 33]. For the evaluation of the Coulomb and exchange integrals (TOLINTEG), tight tolerance factors of 8, 8, 8, 8, 16 were used for all calculations. The reciprocal space of the structure was sampled with a 4×4×6 Monkhorst‐Pack‐type k‐point grid. The starting geometry was taken from experimental data. Both the lattice parameters and atomic positions were fully optimized within the constraints imposed by the space group symmetry. Further on the optimized structure was confirmed to be true local minimum by means of harmonic frequency calculation at Γ‐point. Electronic band structure and density of states (DOS) were calculated. The Brillouin zone path of Γ—X—M—Γ—Z—R—A—Z|X—R|M—A was provided by the web service SeeK‐path [34]. Using the results of the frequency calculation, a theoretical Raman spectrum was calculated by utilizing an analytical CPHF/CPKS scheme (coupled perturbed Hartree Fock/Kohn Sham). The full width at half maximum (FWHM) was set to 8 cm^−1^, the pseudo‐Voigt broadening to 50 : 50 Gaussian:Lorentzian and the laser wavelength to 785 nm. To assign signals in the spectrum to vibrations of the lattice, the software Jmol 14.14.1 [35] was used for visualizing the theoretical vibration modes.

### 2.9 Bond Valence Site Energy (BVSE) Calculations

Li^+^ ion migration pathways in Li_8_TiP_4_ and in Li_7.94_Ti_0.94_Ta_0.06_P_4_ with input structure models obtained from Rietveld refinement and single crystal structure determination, respectively, were analyzed using bond valence site energy (BVSE) calculations [36] employing the softBV bond valence parameter set [37]. The site energies of the Li ions are calculated for a dense grid of points with a resolution of 0.1 Å covering the crystal structure using the transferable Morse‐type softBV force field. Li ion diffusion pathways are identified with the regions of low BVSE. BVSE maps with isosurface levels 0.54 eV over global minimum representing an indication of Li ion migration diffusion pathways were constructed and visualized by using VESTA [38].

## 3 Results and Discussion

### 3.1 Synthesis of Li_8_TiP_4_

Phase‐pure samples of Li_8_TiP_4_ which allow to investigate its crystal structure and physical properties were prepared via ball‐mill syntheses as a prestep with subsequent heat‐treatment. After the ball milling step, most probably a disordered variant of Li_8_TiP_4_ in antifluorite type besides large amounts of binary phosphides as intermediate products and unreacted Ti can be detected. The pure ternary phase is formed after the annealing at 973 K. Alternatively, we applied a method of preparation similar to that reported in the publication of Juza and Langer using a high‐temperature synthesis in tantalum crucibles. The products of the latter reactions contained small amounts of tantalum which was found after structure determinations on single crystals. As the main byproduct from these preparations, TaP was found. Obviously, when using Ta crucibles, the container material may participate significantly in high‐temperature reactions including P, and products of such reactions must be considered critically with respect to the presence of Ta. In fact, Ta had earlier been found as an unintentional contamination [39].

### 3.2 Crystal Structure Determination of Li_8_TiP_4_

**FIGURE 1:**
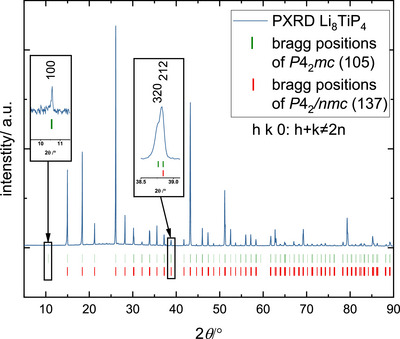
PXRD of Li_8_TiP_4_ with the Bragg positions of the space group P4_2_mc. Two reflections are highlighted by increased magnification, which do not fulfill the extinction condition for hk0: h+k = 2n of P4_2_/nmc, giving evidence for the noncentrosymmetric space group.

**FIGURE 2:**
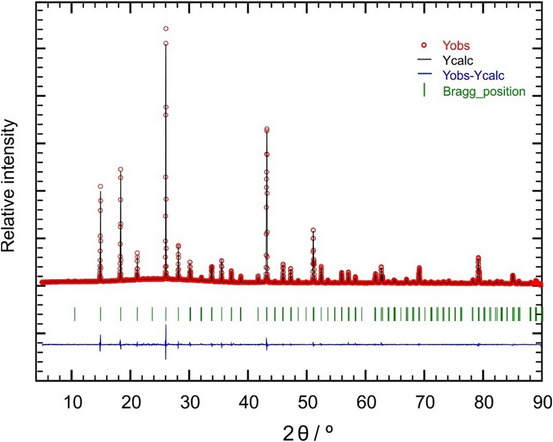
Result from Rietveld refinement of a diffraction pattern of Li_8_TiP_4_ in space group *P*4_2_ *mc* measured with Cu *K_α_*_1_ radiation. The measured reflections are represented by red circles, the fit curve is shown as black line, green stripes mark the Bragg positions and the blue line gives the difference between measured reflections and the fit curve.

The crystal structure of Li_8_TiP_4_ was solved *ab initio* by powder methods and further developed by Fourier analysis [23, 40]. The space group of this early structure solution is *P*4_2_/*nmc* (no. 137), where the P atoms form a distorted cubic close packing with Ti and Li atoms in all tetrahedral voids, the latter also partially occupying the octahedral voids. The voids are filled in an ordered manner so that the structure model contains isolated TiP_4_ tetrahedra. The refinement result for this structure can be found in the supporting information. Although this structure model fits the powder diffraction pattern quite well, a closer look to the diffraction pattern reveals minor discrepancies. In space group *P*4_2_/*nmc* the reflection condition for *hk*0 is *h*+*k* = 2*n*. As can be seen in Figure 1 at least two well defined reflections violate this particular restriction, namely the 100 reflection and the 320 reflection. Therefore, we performed a *translationsgleichen* symmetry descent to the noncentrosymmetric subgroup *P*4_2_ *mc* (no. 105) which is in best agreement with all our experimental findings starting with the presence of abovementioned reflections 100 and 320, and which was confirmed by the results of the structure determination from (Ta containing) single crystals. In Figure 2 the result of the *Rietveld* refinement is shown. The sample is crystallographically phase pure, that is no side phases are detectable by PXRD within the resolution limit of the experiment. With respect to the symmetry relation it seems possible that a HT phase with the centrosymmetric space group *P*4_2_/*nmc* or even with complete Li and Ti cation disorder, possibly with cubic space group *Fm* $\bar{3}$ *m*, exists, particularly with knowledge of the formerly reported antifluorite‐type compounds in the Li/Ti/P system [1, 2, 3, 14, 15]. However, in our investigations no experimental proofs were observed so far. Attempts to quench samples from the annealing temperature resulted in the noncentrosymmetric phase or in mixtures of LiP and TiP, indicating the decomposition of Li_8_TiP_4_. Also DSC analyses did not provide any hint for such a phase transition.

**FIGURE 3:**
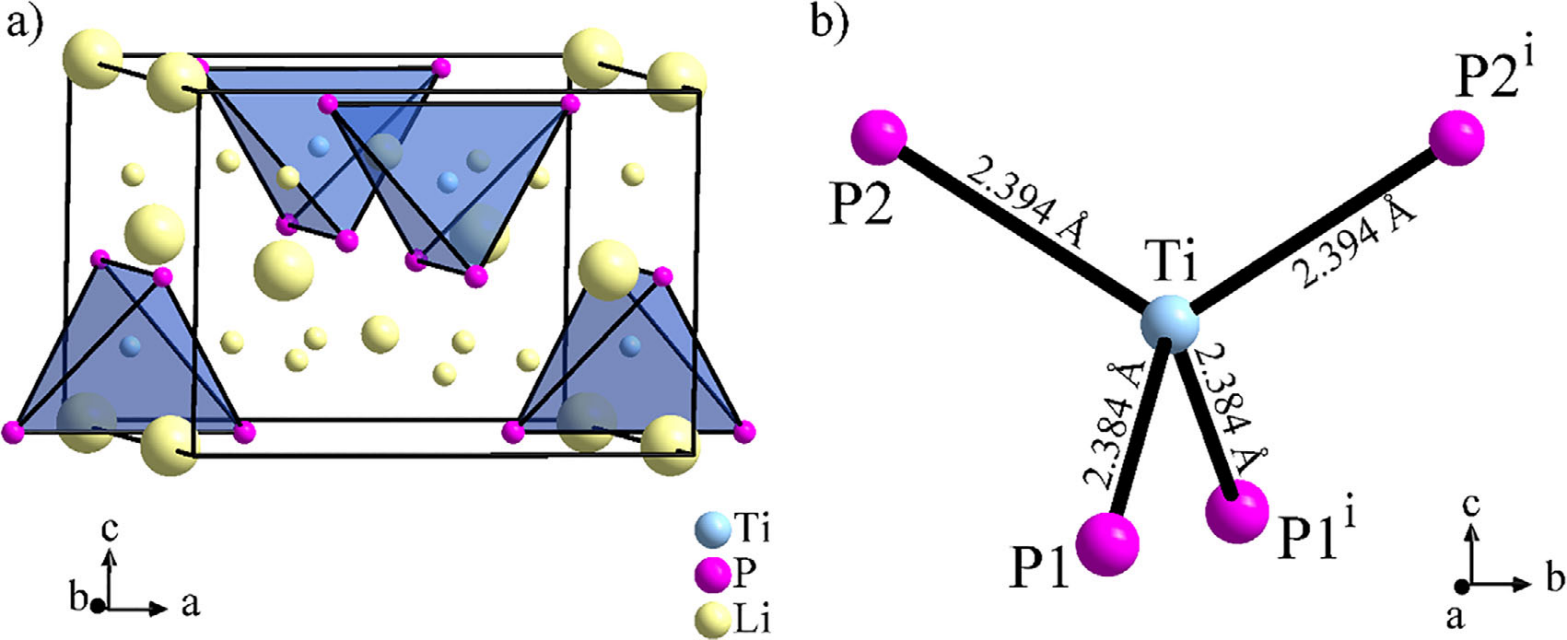
Crystal structure of Li_8_TiP_4_, results from Rietveld refinement of the phase pure compound, (a) unit cell, (b) TiP_4_ anion with bond lengths. All atoms are drawn at 80% probability level.

The final structure in *P*4_2_ *mc* (Figure 3) is similar to the *ab initio* structure model in *P*4_2_/*nmc* and shows slight but significant differences. As the main building principle, the P atoms form a distorted *ccp*, also known from related lithium phosphidotetrelates and ‐trielates. Ti occupies 1/8 of the tetrahedral voids in an ordered manner, leading to isolated TiP_4_ tetrahedra. The remaining tetrahedral voids and parts of the octahedral voids are occupied by Li atoms. As an interesting difference between the two space groups, the single position of the P atoms in *P*4_2_/*nmc*, an 8 *g* site, splits into two positions at 4*d* and 4*e* in *P*4_2_ *mc*, which results in two different Ti‐P distances of 2.384(6) Å and 2.394(6) Å as well as different Li surroundings for the two independent P atoms. This is in accordance with the two phosphorus signals observed in the ^31^P NMR spectra, which best can be explained by splitting the phosphorus position into two distinct Wyckoff sites (see below in the NMR‐section). In detail, P1 is in distorted cubic environment of one Ti and seven Li atoms in the surrounding tetrahedral voids with distances up to 2.76(2) Å, with two next closest Li atoms occupying octahedral voids in a distance of 3.34(1) Å. In contrast, for P2 the pseudo‐cubic surrounding contains two empty tetrahedral voids besides one Ti and five Li atoms, but three Li atoms in octahedral voids are in comparable distances below 2.79 Å with another one in a distance of 3.28(1) Å. Further P‐Li distances are above 4.5 Å, P‐Ti above 4.9 Å. In summary, P1 is in a 1(Ti)+7+2, P2 in a 1(Ti)+8+1 coordination.

The lengths of the Ti‐P bonds in Li_8_TiP_4_ are relatively short compared to those in related compounds, which often show higher coordination numbers for both elements [41, 42, 43]. Quite few shorter bonds are reported so far, e. g. in the binary Ti_2_P (2.321 Å) representing another example of a compound containing a tetrahedral coordination of Ti by P atoms [44]. Isolated TiP_4_ tetrahedra, to the best of our knowledge, are known only from four different compounds so far, namely Li_9_TiP_4_, Li_11_TiP_5_, Sr_4_TiP_4_ and Ba_4_TiP_4_ [14, 15, 17]. The former two are hard to compare in terms of Ti‐P bond‐lengths, as there are only mixed positions with statistical distribution of lithium and titanium, but for the latter two the shortest Ti‐P bonds are 2.379 Å and 2.402 Å, respectively [17], matching very well the average value of 2.389 Å for the title compound. This short bond‐length is indicative for a covalent interaction between titanium and phosphorus.

**FIGURE 4:**
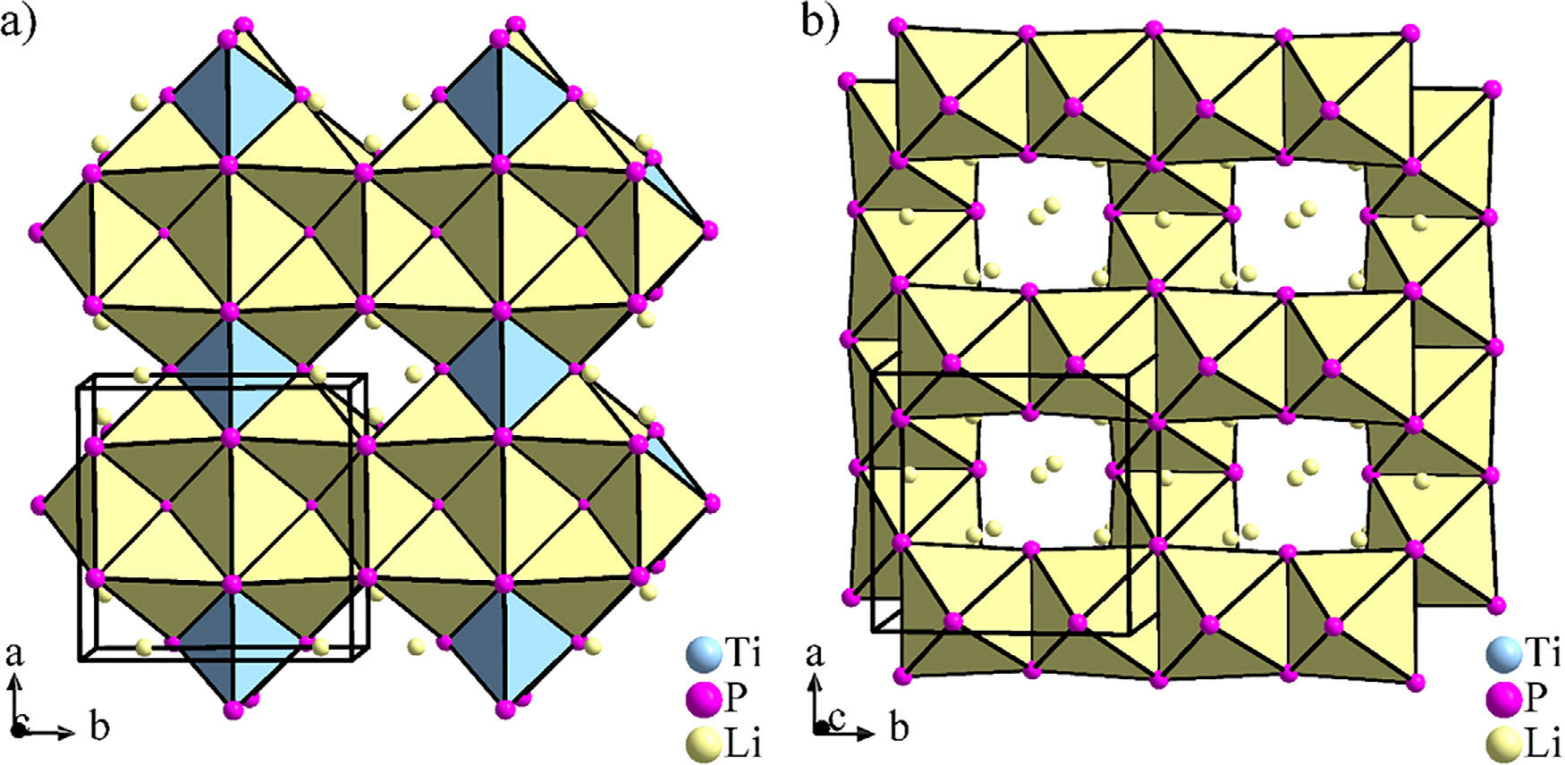
Crystal structure of Li_8_TiP_4_, occupation of: (a) tetrahedral and (b) octahedral voids of the distorted ccp of P atoms. Blue tetrahedra are centered by Ti, yellow polyhedra by Li cations.

The crystal structure model contains four crystallographic sites for the Li atoms. Basing on the *ccp* formed by the P atoms, these are located in tetrahedral voids at 2*c* (Li1), 8*f* (Li2), and 2*b* (Li3) and in an octahedral void at a 4*d* site (Li4), see Table 1. Full occupation of these sites would result in the expected composition Li_8_TiP_4_. Within this packing, 1/16 of the tetrahedral voids, centered along the *c* axis (Figure 4.a), and 1/2 of the octahedral voids (Figure 4.b) remain unoccupied. During the refinement of the powder data, site occupations slightly lower than 100 % for all Li positions were found, accompanied by small amounts of residual electron density in the remaining voids. However, attempts to refine additional Li atom positions with low occupations by using the X‐ray powder data did not converge. The refinement results including the lower Li site occupations, and thus an unbalanced chemical composition, are shown as supporting information. Li‐P distances range from 2.49(2) Å to 2.76(2) Å for Li cations in tetrahedral voids, while the Li4 atom in the octahedral void is heavily off‐centered in direction of one octahedron face and showing three shorter (2.624(8)–2.78(2) Å) and three longer Li‐P distances (3.28 (2)–3.34(2) Å), indicating the too large size of the void for a Li^+^ cation.

**FIGURE 5:**
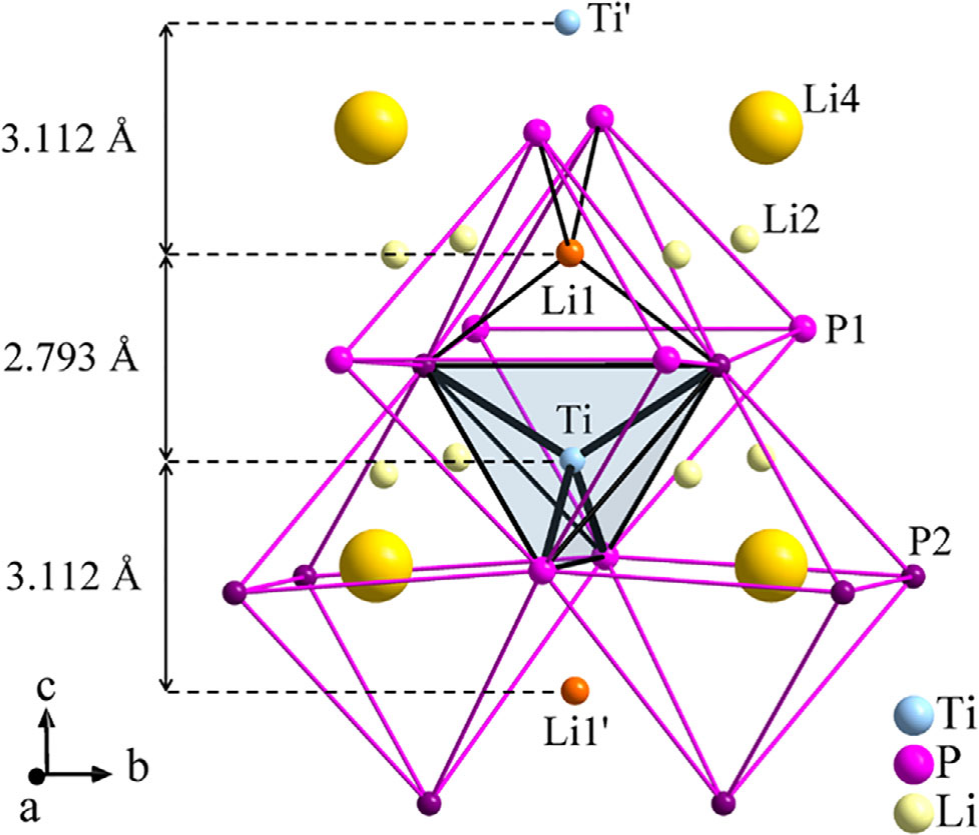
Section of the crystal structure of Li_8_TiP_4_, shown are the four octahedral voids (pink lines) adjacent to the Ti‐filled tetrahedral void (blue tetrahedron), two of them are filled with Li4 ions, two are empty. As a consequence of this filling scheme, the Ti and Li1 cations are slightly shifted in direction of the empty voids and the Ti‐Li1 distances alternate as shown left. All atoms are drawn at 80% probability level, the crystallographically independent Li cations are drawn as orange (Li1), light yellow (Li2) and dark yellow (Li4) spheres, respectively, the P2 atoms are drawn in darker pink than the P1 atoms.

According to this structure model, some structural features can be identified which are significantly violating the higher symmetric space group *P*4_2_/*nmc* and thus are structural arguments for the assignment of space group *P*4_2_ *mc*: The presence of two positions for the P atoms in *P*4_2_ *mc* provides more degrees of freedom for the TiP_4_ tetrahedron. The P2‐Ti‐P2’ angle of 115.7° is notably larger than the remaining five P‐Ti‐P angles (108.1°‐108.6°), and the same is valid for the corresponding P2‐P2’ edge length of 4.054 Å compared to the five nearly equal P‐P edge lengths (3.868‐3.871 Å). This pronounced elongation can be attributed to the adjacent Li1 cation, which is located in close proximity to the respective tetrahedral edge. To maintain the typical Li─P separations of 2.53(3) Å, this edge has to be elongated. The Li1 ions occupy tetrahedral voids that share two opposite edges with the TiP_4_ tetrahedra. As a result, TiP_4_ and Li1P_4_ tetrahedra are connected via common edges, forming a chain of edge‐sharing tetrahedra propagating along the *c* axis. It is noteworthy that the Ti─Li1 distances along this row alternate (Figure 5). The shorter Ti─Li1 separation represents by far the shortest Ti─Li contact (2.793 Å) within the structure, whereas the longer distance is comparable to the remaining Ti─Li separations (3.112 Å). The reason for the alternating Ti─Li1 distances is probably the mutual electrostatic repulsion with the Li4 cations located in two of the neighbouring octahedral voids. As a consequence, Ti and Li1 ions are slightly shifted towards the unoccupied octahedral vacancies. The occurrence of these different Ti─Li1 distances provides additional evidence for the space group P4_2_mc, since in P4_2_/nmc the Ti and Li1 sites would occupy special positions and therefore exhibit identical Ti─Li separations. Notice, that the occupation pattern of the octahedral voids observed here can also be realized with SG P4_2_/nmc.

### 3.3 Single Crystal Structure of Li_7.9_Ti_0.9_Ta_0.1_P_4_ and Compositional Phase Width of Li_8_TiP_4_

Preliminary experiments in the Li/Ti/P system aiming to obtain Li_8_TiP_4_ were performed in tantalum crucibles at high temperatures. Single crystals suitable for X‐ray structure determination were successfully isolated from one of these syntheses. Same as the Li_8_TiP_4_ powder structure, the space group of the crystals was determined as *P*4_2_ *mc*, and the structure was refined as inversion twin with a Flack parameter close to 0.5. The equal amounts of the two possible domain orientations suggest a transition from higher to lower symmetry, however, also from the single crystal investigations no hints for the thermal or synthetic conditions around this possible transition are observed to date. During the structure determination it turned out that this phase contains a significant amount of Ta as well as that the Li content was lower with respect to pure Li_8_TiP_4_. According to compounds which have been obtained at similar experimental conditions [39, 45], tantalum can be assumed to be in oxidation state +5. To maintain charge balance, one Li atom is removed for each Ta that substitutes for Ti in the crystal structure (see below) resulting in the general formula Li_8−_ *_x_* Ti_1−_ *_x_* Ta*_x_* P_4_, which is consistent with the experimental findings.

**FIGURE 6:**
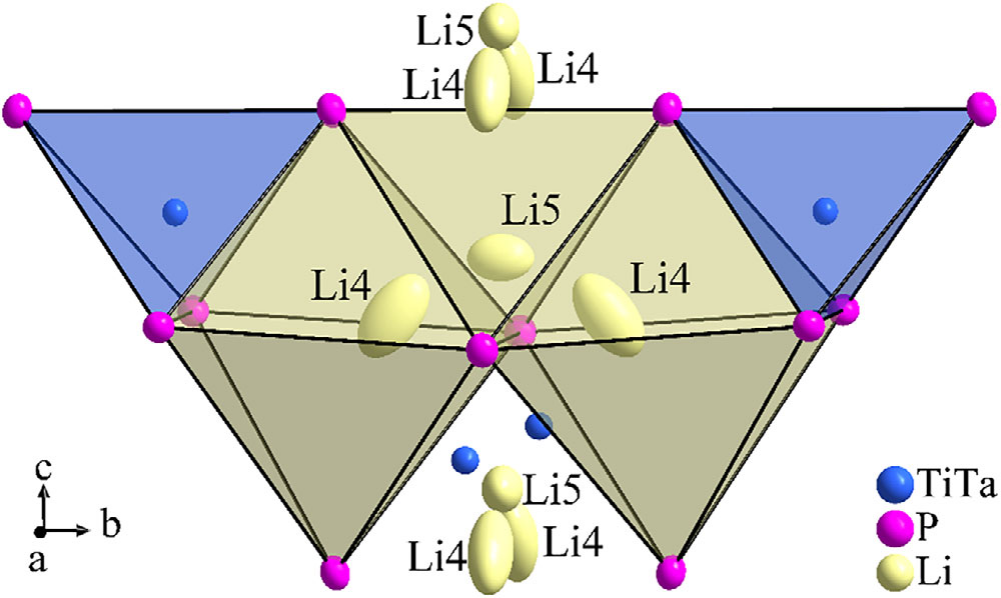
Section of the crystal structure of Li_8−x_Ti_1−x_Ta_x_P_4_, shown is the environment of the Li5 atom in a tetrahedral void with two close Li4 atoms in adjacent octahedral voids. Anisotropic displacement ellipsoids of all atoms are drawn at 80% probability level, polyhedra built from P atoms around the Li and Ti/Ta atoms are shown in yellow and blue, respectively.

Despite the slightly different chemical composition, the structure type is retained, and the single crystal data confirm the structure model obtained from powder diffraction analysis of phase‐pure Li_8_TiP_4_. Ta atoms occupy the Ti position as can be expected for Ti^4+^ and Ta^5+^ cations with almost the same radii [46]. Consequently, the Ti/Ta‐P distances are very similar to Ta‐P distances with Ta in tetrahedral coordination by P atoms [39, 45, 47, 48, 49]. The mean Ti/Ta‐P bond length is almost identical compared to Li_8_TiP_4_ but shows a slightly larger variation (2.3790(8) Å and 2.4050(7) Å), while the P‐Ti/Ta‐P angles are more regular. Short and long Ti/Ta‐Li1 distances along the *c* axis, though with a smaller deviation (2.898(13) Å and 3.022(13) Å), are observed as well. This is obvious, as the extremely short distance between the Li1 and the *TM* cations should react particularly sensitive to the higher mean charge of the transition metal atoms (as well as the lower occupation of the octahedral void, see below). A striking difference between the two structure models is the localization of an additional Li5 atom at the remaining unoccupied tetrahedral void in Li_8_TiP_4_ with a low but significant occupation of 6.37(6) %, combined with a reduced occupation of Li4 in the octahedral void of 93.63(6) %. No further atom position, for example at the empty octahedral void, was observed. The occupancies of Li1, Li2, and Li3 atoms are not affected and remain close to 100 % at free refinement, and were thus fixed to full occupation in the final refinement cycles. Substitution of Ti^4+^ by Ta^5+^ reduces the overall lithium content for a charge balanced compound, and, although another additional Li atom appears at the tetrahedral Li5 site on Wyckoff position 2*a*, this net loss of Li is completely compensated by the decrease of the occupation at the octahedral Li4 site (4*d*). The Li5 position lies in close proximity to two Li4 atoms, and the main extension axes of the displacement ellipsoids of these atoms indicate parts of a possible path for the Li ion conduction movement (Figure 6). As another consequence of the short distance between Li5 and Li4, each occupied Li5 atom site blocks the occupation of both the neighboured Li4 atom sites. A list of bond lengths is given as supporting information. The presence of some partial atom occupations at a position analogous to the Li5 position in pure Li_8_TiP_4_ cannot be excluded, however, it was not possible to refine a stable atom at this site from powder data.

An interesting aspect of this structure type is the ability to host variable amounts of Li. This indicates the possibility of the existence of a phase width, at least if charge balance is governed by another (transition) metal of similar ionic radius.

### 3.4 Structure Relations Among Corresponding Lithium Tetrel Phosphides

**FIGURE 7:**
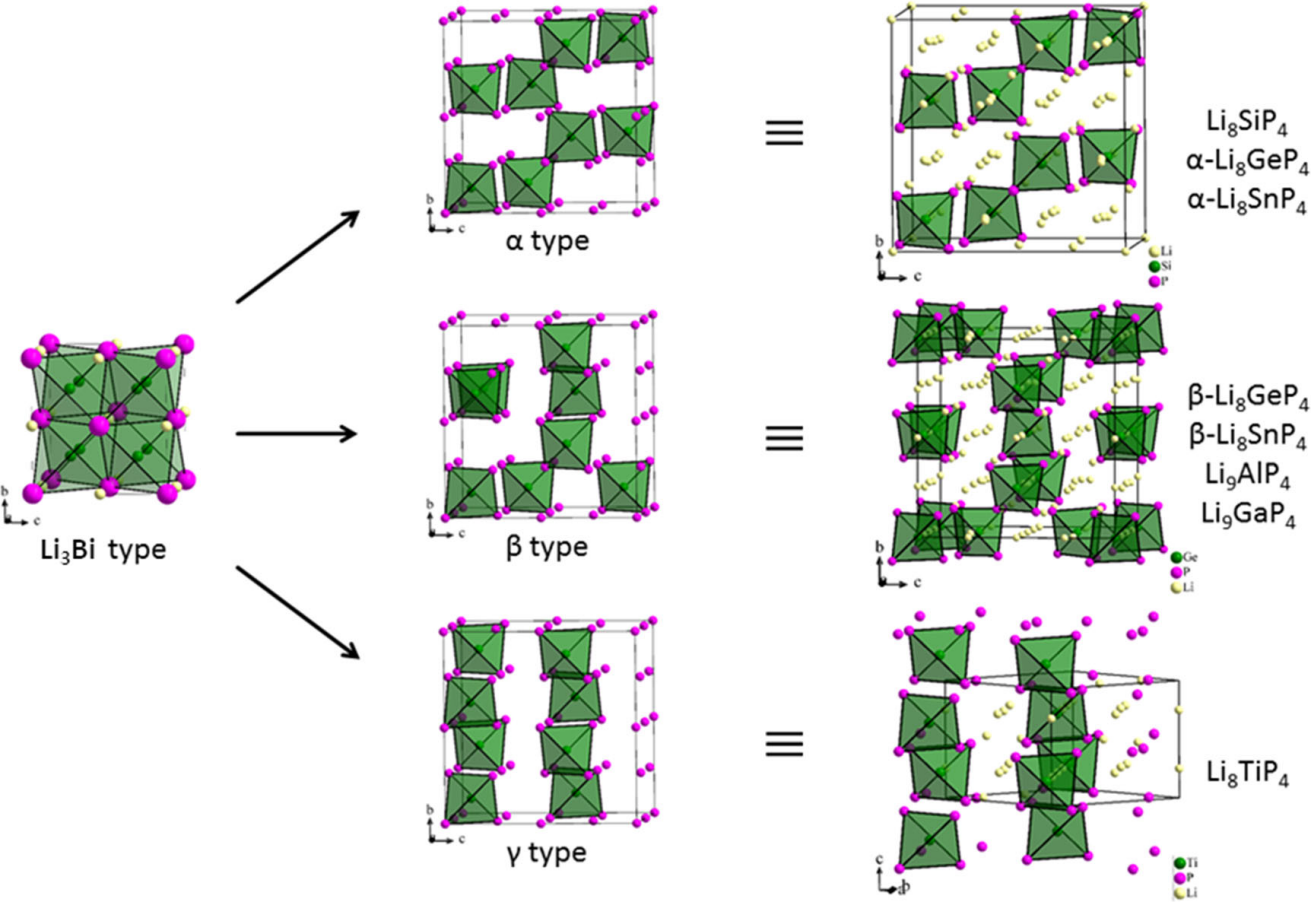
Ordered structure types of the Li_8_ *Tt*P_4_ and related Li_9_ *Tr*P_4_ compounds, left: aristotype of the different structures; middle: 2×2×2 unit cell of Li_3_Bi with the differently ordered packing of *Tt*P_4_ tetrahedra in the three structure types; right: structure types with correct setting resp. unit cell. Pink spheres: ccp forming atoms (Bi in Li_3_Bi type; P in α, β, and γ types); yellow spheres: Li atoms; green spheres: *Tt* or Ti atoms (in Li_3_Bi: Li in tetrahedral voids).

**FIGURE 8:**
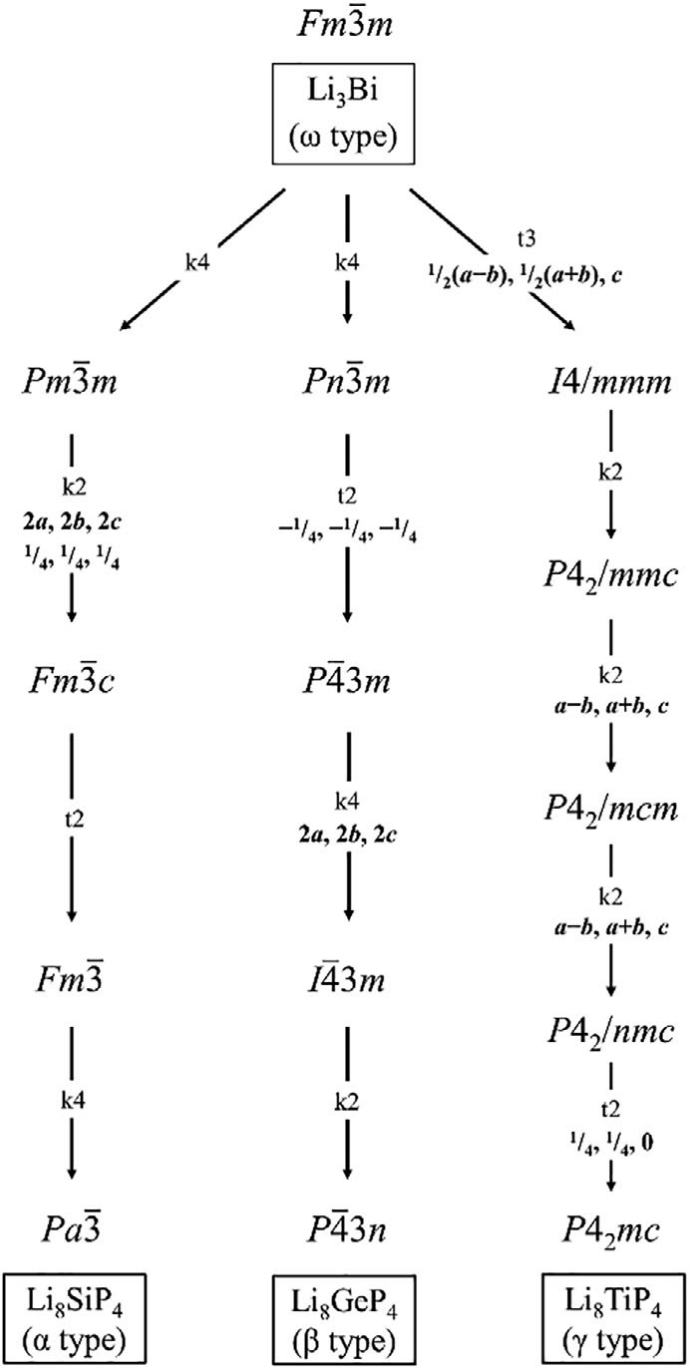
Symmetry degradation from the Li_3_Bi structure type to the three different ordered structure types so far observed for the Li_8_ *Tt*P_4_ compounds.

Li_8_TiP_4_ represents the transition metal analogue of the known lithium phosphidotetrelates Li_8_SiP_4_, Li_8_GeP_4_, and Li_8_SnP_4_. Remarkably, although main building principles are the same, the crystal structure differs. For the lithium group‐14 phosphides Li_8_ *Tt*P_4_, two structure types labeled as α and β structure are known, which correspond to the space groups *Pa* $\bar{3}$ and *P* $\bar{4}$3*n*, respectively. Both polytypes represent a distorted 2×2×2 superstructures of the antifluorite type with partly occupied octahedral voids, or—as a chemically more appropriate description—of the Li_3_Bi structure type as defect variants with reduced occupation of the octahedral voids. The P atoms form a *ccp* where the respective tetrel atoms occupy 1/8 of the tetrahedral voids and lithium ions fill the remaining tetrahedral voids as well as a part of the octahedral voids. The distinction between the α and β structure types arises from the arrangement of the tetrel‐phosphide tetrahedra in the unit cell. To date, Li_8_SiP_4_ is known exclusively in the α form, whereas Li_8_GeP_4_, and Li_8_SnP_4_ adopt both the α and β modification, respectively [5, 8, 9]. Interestingly, the related, triel element containing compounds Li_9_AlP_4_ and Li_9_GaP_4_ comprising slightly higher amount of Li atoms adapt the β form [50, 51]. In order to compare the arrangement of TiP_4_ tetrahedra in Li_8_TiP_4_ the unit cell must be expanded to form a pseudo‐cubic supercell ($a^{\prime }=\sqrt{2}\;\cdot a,\;b^{\prime }=\sqrt{2}\;\cdot b,\;c^{\prime }=2\cdot c)$. As illustrated in Figure 7, the Li_8_TiP_4_ shows a new arrangement of tetrahedra, thus labelled as γ type. Most interestingly, although identical in composition, these structures are not only different in the ordering of *Tt*P_4_ tetrahedra, but exhibit no other symmetry relationship—such as group/subgroup relationship—apart from their connection by the Li_3_Bi aristotype (Figure 8). This is even more remarkable, as for Li_8_GeP_4_ and Li_8_SnP_4_ a thermal phase transition between α and β types is observed, which requires a position change of the *Tt*P_4_ tetrahedra.

#### 3.4.1 ^31^P and ^6^Li MAS NMR Spectroscopy

**FIGURE 9:**
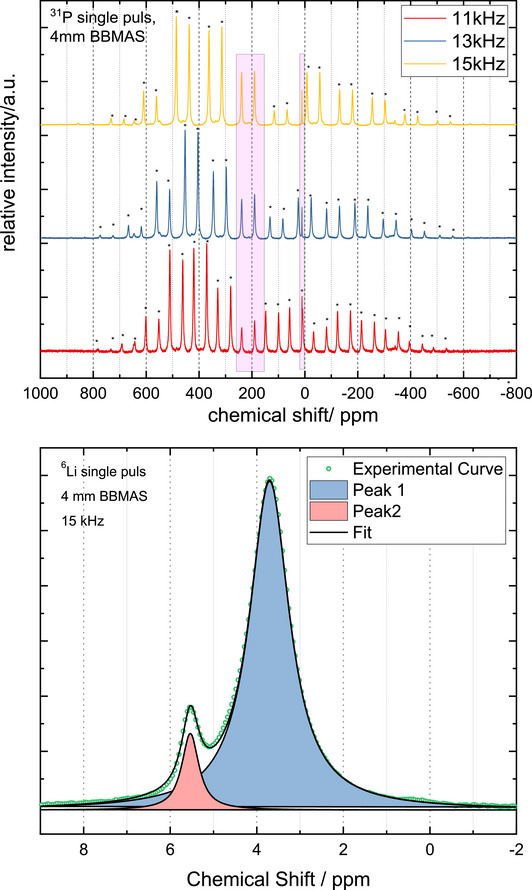
MAS NMR spectra of ^31^P (top) and ^6^Li (bottom).

The ^31^P NMR (Figure 9) shows, besides many rotation side bands two distinct phosphorus signals in the ratio 1:1 (239, 190 ppm) revealing at least two different phosphorous atoms with slightly differing chemical environments. Known that ^31^P NMR resonances typically span a wide chemical‐shift range, the values observed here therefore fall within the range reported for chemically related compounds such as Li_7_TaP_4_ with two signals at 158 and 189 ppm [55]. This finding supports also the correct choice of the lower symmetric space group *P*4_2_ *mc* (no. 105) with two different crystallographic sites (4*d*, 4*e*) for the P atoms. Therefore, space group *P*4_2_/*nmc* (no. 137) with only one P atom position can be excluded, because only one signal in the ^31^P NMR spectrum should be present. The low field shift in comparison to the analogue lithium group‐13 and group‐14 phosphides arises from a strong de‐shielding as a result of a more covalent Ti─P bond. In the ^6^Li spectrum (Figure 9) two overlapping signals (3.72 and 5.54 ppm) are observed with the ratio of 7/1, indicating a different chemical environment for one of the eight present Li atoms, and a less dynamic behavior compared to the remaining Li atoms leading to the smaller half width of the smaller signal. While crystallographically two Li sites, Li1 (2*c*) and Li3 (2*b*), represent one eighth of the Li atoms, respectively, most likely the Li1 atom should cause the isolated signal due to two reasons: first Li1 is lying closest to the Ti^4+^ ion which should cause a deshielding and, thus, a low‐field shift, and, moreover Li1 shows the lowest tendency to participate in the mobile behavior of the Li atoms according to the BVSE calculations so the sharpest signal for Li atoms should be found here.

### 3.5 Raman Spectroscopy

**FIGURE 10:**
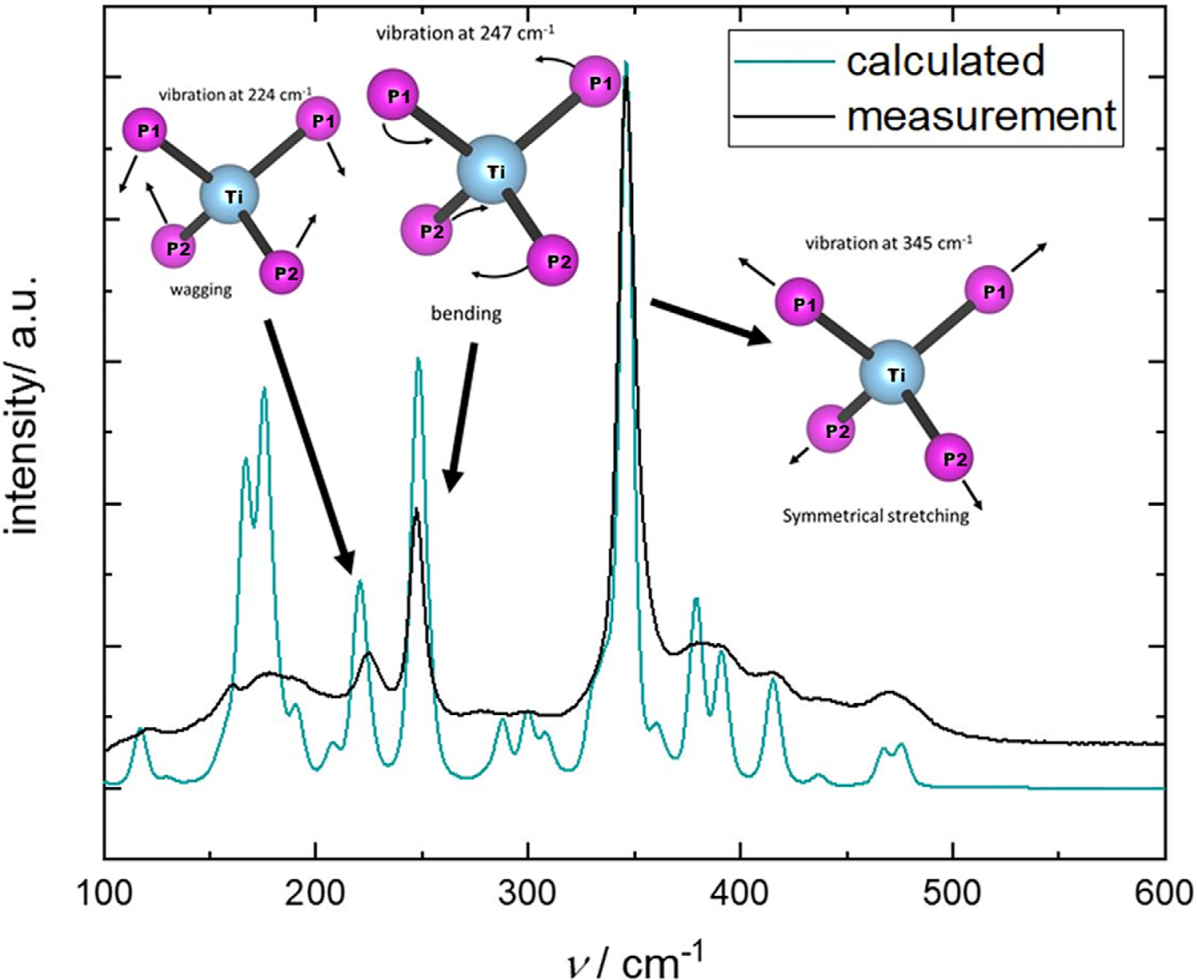
Experimental (black) and calculated (turquoise) Raman spectra for Li_8_TiP_4_. The wavenumbers of the calculated Raman spectrum have been multiplied by a factor of 0.94 to better match the highest intensity modes of the experimental spectrum.

The Raman spectrum of Li_8_TiP_4_ shows several sharp bands below 500 cm^−1^ (see Figure 10). To assign the measured bands to vibrational modes in the structure, a theoretical spectrum has been calculated based on DFT‐PBE0/TZVP and the wavenumbers have been scaled by a factor of 0.94 to match the signals. The model for the calculated spectrum was taken from the crystal structure. Minor adjustments had to be done to the lithium ordering in the structure so only fully occupied positions existed in the input file. The strongest band originated from the symmetrical stretching of the TiP_4_ tetrahedra. Other strong signals represent bending and wagging modes of the tetrahedra, which in accordance with the short bond length supports the proposal of actual covalent binding between phosphorus and titanium atoms. A list of measured modes and their assignments based on the DFT calculations is given as supporting information.

### 3.6 Electronic Structure

**FIGURE 11:**
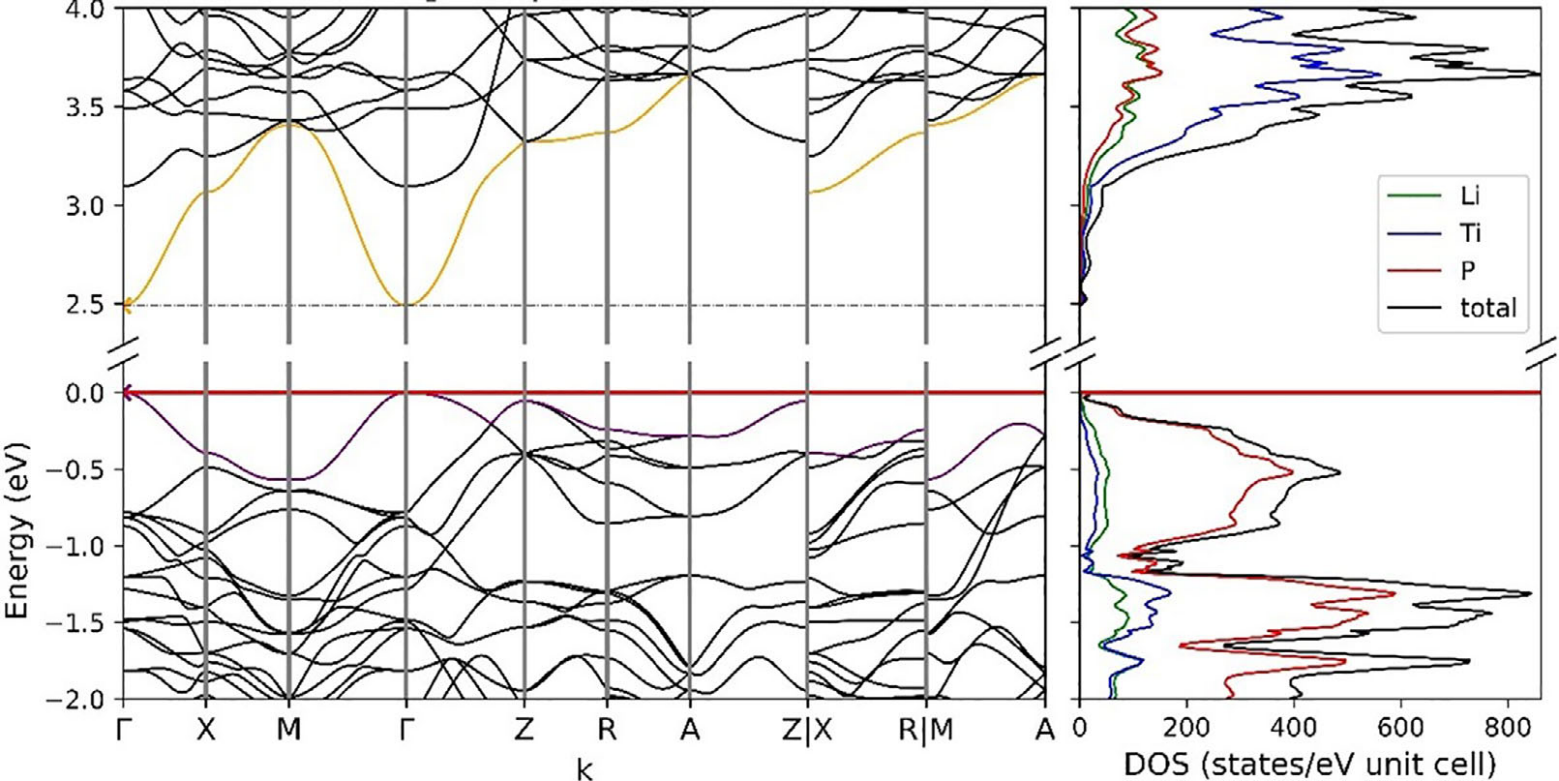
Band structure and density of state for Li_8_TiP_4_ exhibiting a direct bandgap at Γ of 2.5 eV. The fermi level is located at 0 eV.

Li_8_TiP_4_ is a semiconductor with a direct bandgap of 2.5 eV at Γ (see Figure 11), matching the dark red color of the crystals. Valence and conduction bands show a moderate dispersion, which can be contributed to the rather localized chemical bonds of the TiP_4_ tetrahedra. Below the Fermi level P atom contributions to the DOS are most dominant, while the conduction bands mostly originate from Ti atom states. Similar to recent studies, the former can be attributed to the lone pairs of the phosphorus atoms [52]. A Mulliken population analysis further on revealed covalent interactions between Ti and P within the isolated tetrahedra and mostly ionic interactions towards the Li cations (Table S14 Supporting Information).

### 3.7 Electrochemical Characterization

The conductivity mechanism in the lithium phosphidotetrelates and ‐trielates is well investigated and understood. In the distorted *ccp* of P atoms the octahedral voids are interconnected via common edges, same as for the interconnection of the tetrahedral voids. All faces of the octahedral voids are shared with adjacent tetrahedral voids and vice versa. It has been shown, that the most favorable diffusion path for lithium within these structures is via face sharing octahedral and tetrahedral voids, however, if this path is not accessible, diffusion via edge sharing tetrahedral voids is also observed [5, 6, 7, 8, 9, 10, 12].

**FIGURE 12:**
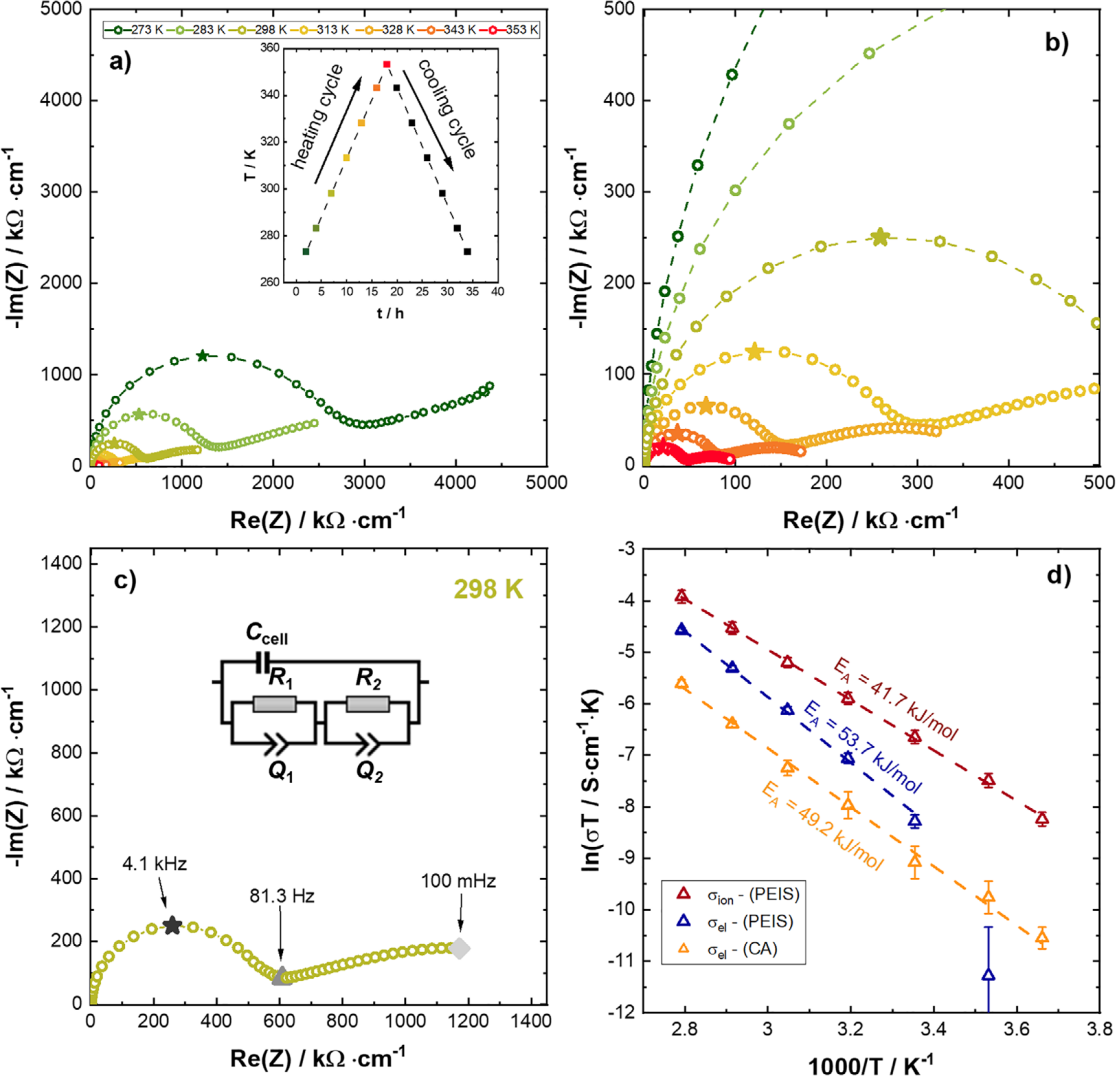
(a) Nyquist plots of Li_8_TiP_4_ to determine the activation energy with spectra collected at temperatures between 273 and 353 K using ion blocking electrodes. The color coding corresponds to the applied temperature (from green: 273 K to red: 353 K). The inlet shows the applied temperature profile with the corresponding color coding. (b) Zoom in of the Nyquist plot to resolve the high temperature impedance responses of Li_8_TiP_4_, using the same color coding for the temperature assignment. Mark with filled asterisk symbols are the apex‐frequencies of the *R*/*Q* element for the corresponding first semi‐circle. (c) Nyquist plot for 298 K to better illustrate the impedance at room temperature with the frequency assignment and the applied equivalent circuit model which was used for the fitting of the impedances for all temperatures. All impedance spectra are normalized to the sample thickness *t*_sample_. (d) Corresponding Arrhenius plots of the product of temperature and conductivity (*σ*_Li_∙*T*) obtained in the heating as well as in the cooling branch for both, the fitted first semi‐circle (red dots) as well as for the second semi‐circle (blue dots). Additionally, to the product of conductivity value calculated from the temperature dependent PEIS‐measurements, we also collected temperature dependent CA measurements to calculate the activation energy for the pure electron transport (yellow triangles). The error bars for each measurement is based on the standard deviation from three independent measurements. To calculate the activation energy *E*_a_ the linear fit through both branches was used.

Experimentally, the Li^+^ ion conductivity of Li_8_TiP_4_ was investigated using potential electrochemical impedance spectroscopy (PEIS) using an ion‐blocking stainless‐steel electrode configuration [6]. The resulting impedance responses measured for temperatures between 273 and 353 K are shown in Figure 12.a,b using a heating up color coding. Independently from the temperature, each spectrum shows a semi‐circle in the high frequency MHz/kHz‐regime (see marked frequency points) with apex frequencies between 2.0 kHz for 273 K and 27.6 kHz for 353 K and a more or less pronounced onset for a second semi‐circle in the low frequency mHz‐regime. Especially for higher temperatures (343 and 353 K) the low frequency semi‐circle is almost fully resolved (see Figure 12.b).

Typically, such nondeformed semi‐circles, resolved here for the higher frequencies, can be described as parallel circuit element of a resistor and a constant phase element (*R*_1_/*Q*_1_, cf. Figure 12.c). To account for the stray capacitance of the used cell setup, the impedance data were fitted with an additionally fixed capacitance *C*_cell_ of 1.83 × 10^−10^ F in parallel to the *R*_1_/*Q*_1_ elements [53]. However, as recently discussed, this considered cell stray capacitance does not affect the determined ion transport properties of the sample. For the constant phase element, corrected by the cell stray capacitance, the fit of Li_8_TiP_4_ collected at 298 K results in an *α* value of ≈ 0.98 and a *Q* parameter of ≈ 3 × 10^−10^ F∙s^(^ *^α^* ^−1)^. Hence the *α* value is reasonably close to 1, the *Q* parameter becomes essentially equivalent to a pseudo‐capacitance for the high‐frequency semi‐circle. From the resistances *R*_1_ of the high frequency semi‐circle (obtained from three independently measured cells), a conductivity *σ*_1_ can be calculated, resulting in values of (4.3 ± 0.6) × 10^−6^ S∙cm^−1^ at 298 K. As previously discussed in publications of our group [6, 9, 13, 54], the ionic conductivity *σ_1_*, calculated from the resistance *R_1_*, represents the total ionic conductivity and is including all parts of the conductivity, such as bulk‐contribution, grain‐boundary‐contribution, and eventually contact resistances of the cell setup. Thus, enabling the estimation of the lower limit of the ionic conductivity of Li_8_TiP_4_. The main contribution to the described resistance is the grain boundary contribution, since even higher frequencies (> 10 MHz) [54] are required to resolve the bulk conductivity, and therefore resolution at room temperature is not possible in the evaluated conditions and cell setup.

As different to recently reported structurally similar solid electrolytes, additionally to the high frequency semi‐circle, all impedance spectra showing an onset of a second semi‐circle instead of a low frequency tail. This low frequency part (≈ Hz/mHz‐region) indicates a second transport phenomenon. By fitting of a second *R*_2_/*Q*_2_ element to the Hz/mHz‐region, a conductivity of (8.5 ± 1.0) × 10^−7^ S∙cm^−1^ can be calculated from *R*_2_ for the second semi‐circle at room temperature (light green spectrum, Figure 12.c), for the second transport phenomena. However, it should be mentioned, that this value is strongly affected by the rather low alpha value of *α* ≈ 0.5 at 298 K and a *Q* value of 6 × 10^−6^ F∙s^(^ *^α^* ^−1)^ (298 K) as well as the not completely resolved semi‐circle for the lower temperatures resulting in unreliable fits at low temperatures (cf. Figure S10b). We would therefore like to clarify at this point that an interpretation of a second transport phenomenon‐based solely on impedance spectroscopy data would not be robust and that extrinsic effects (e.g., surface contamination, impurity phases) could also contribute. In order to make a more precise statement about the electronic conductivity and to further elucidate the second transport phenomena, we used CA measurements in the same cell setup to determine a potential electronic conductivity by applying different DC polarizations (50 mV, 100 mV, and 150 mV) under ion blocking conditions. Estimated from the polarization measurements the electronic conductivity was calculated to be (3.8 ± 0.9) × 10^−7^ S∙cm^−1^ at 298 K (based on the standard deviation of three cells). The fact that the partial electronic conductivity measured by DC polarization is essentially of the same order of magnitude as the conductivities calculated from the second (low‐frequency) semi‐circle in the PEIS measurements, we attribute the semi‐circle in the Hz/mHz‐region to the electronic transport. Therefore we assume, the first (high‐frequency semi‐circle) can be attributed to a potential Li‐ion conductivity. This is further supported, by the calculated capacitance values of the *R*_1_/*Q*_1_ elements, which is in the order of ≈ 10^−10^ F at 298 K, and consistent with the assumption that the pseudo capacitance *Q*_1_ of the high frequency semi‐circle represents the sum of the intra‐grain and grain boundary Li ion transport (typical ranges for intra‐grain is 10^−12^ F and 10^−9^ F for grain boundary) [9, 53]. Furthermore, from the temperature dependent PEIS measurements between 273 and 353 K, the respective activation energies (*E_A_^PEIS^*) for both transport phenomena were investigated. Independent from the temperature, the above‐described two semi‐circle response can be observed, with temperature sensitivities for both semi‐circles. Following the argumentation from above, the first semi‐circle is in the following attributed to the total Li ion conductivity of Li_8_TiP_4_ for all investigated temperatures_._ As typical for Li ion conductors, the first (high frequency) semi‐circle becomes smaller for higher temperatures (Figure 12.a,b), indicating an increase in the Li^+^‐conductivity. This trend is also observable for the low‐frequency part, however, with a different sensitivity regarding the temperature. Determined from the resistances either for the first semi‐circle resistance *R*_1_ or for the second semi‐circle resistance *R*_2_, the corresponding *σ*∙*T* product was calculated and is shown in the Arrhenius plot as a function of the inverse temperature in Figure 12.d. For both transport phenomena (red and blue values in Figure 12.d) a linear trend is observable in the Arrhenius plots which yield in an activation energy of *E_a,Li_^PEIS^* = 41.7 ± 0.8 kJ∙mol^−1^ (≈ 0.43 eV) for the Li ion conductivity (from the high‐frequency semi‐circles) and an activation energy of *E_a,el_^PEIS^* = 53.7 ± 1.6 kJ∙mol^−1^ (≈ 0.56 eV) for the electronic conductivity (low‐frequency semi‐circles), determined from three independently measured cells using the values of the heating cycle. In this context, it shall be mentioned that the calculated conductivities (and thus the corresponding *σ*∙*T* values) for the heating and cooling branch differ by less than 3 %. The represented error bars are calculated separately for heating and cooling steps by taking the mean value of three independent measurements/cells. However, due to the minor resolved second semi‐circle for the low temperatures of 273 K and 283 K ((dark) green impedance responses in Figure 12.a), the respective resulting fit for the second semi‐circle is significantly worse and, thus, the corresponding *σ*∙*T* value is highly affected. Therefore, we exclude these points for the linear fit and the corresponding calculated activation energy (*E_a,el_^PEIS^*). Additionally, we also collected temperature dependent CA measurements using an ion blocking electrode design. From these measurements we can calculate an activation energy (*E_a,el_^CA^*) for the pure electron transport. The resulting *σ*∙*T* values are plotted as yellow values in the Arrhenius plot (Figure 12.d). From the slope, the activation energy for the electron transport is determined (by CA) to be *E_a,el_^CA^* = 49.2 ± 0.9 kJ mol^−1^ (≈ 0.51 eV). Generally, the *σ*∙*T* values determined by CA measurements are slightly lower compared to the *σ*∙*T* values determined by fitting the second semi‐circle in the PEIS response. Therefore, the conductivity is over‐estimated by fitting the second semi‐circle from the PEIS measurements. However, the calculated activation energy by CA (*E_a,el_^CA^*) is slightly lower compared to the activation energy given by the PEIS measurements (*E_a,el_^PEIS^*). Hence, a comprehensive electrochemical investigation of Li_8_TiP_4_ is beyond the scope of this study, a final attribution of the semi‐circles in the PEIS‐measurements to Li‐ion and electron transport is difficult. Furthermore, since the electron and ion conductivity are in the same order of magnitude, we assume that the ionic conductivity is in the same region or lower as the electron conduction.

### 3.8 Bond Valence Site Energy (BVSE) Calculations

**FIGURE 13:**
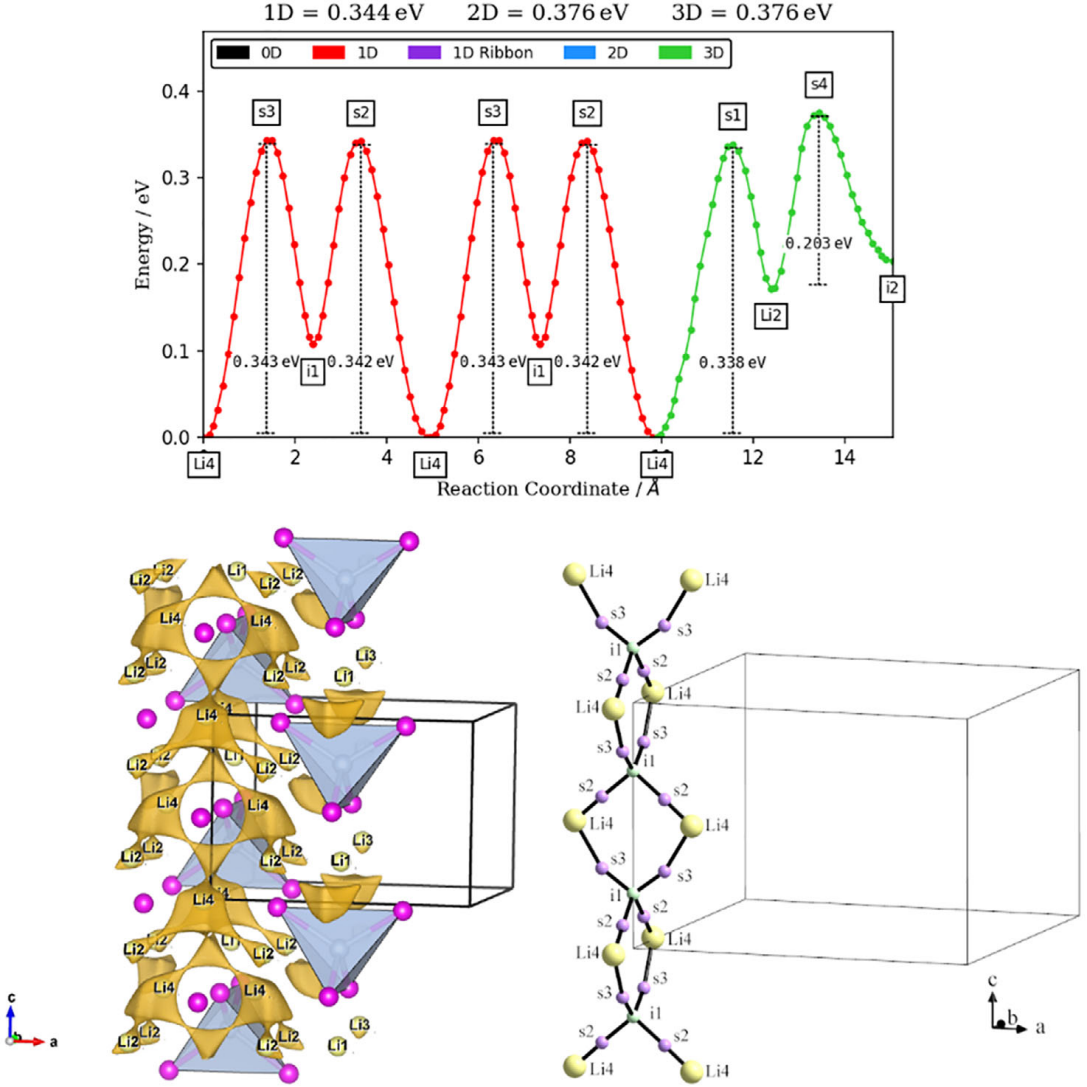
Lowest migration barriers for Li_8_TiP_4_ and the resulting isosurface and 1D pathway along the c axis.

BVSE maps generated following the softBV method [36] by applying structural information were used to detect the most probable ion diffusion pathways in the crystal lattice. This method can provide a first idea of the diffusion mechanism within the structure; however, it bases on the static structure model without taking any dynamic contributions including phonons into account so its validity is limited and should rather be considered as a rough estimation. Also, only ionic interactions are regarded, while in Li_8_TiP_4_ we clearly observe covalent Ti─P bonds, which will influence the result. With respect to these limitations, an interesting result of our calculations is, that in Li_8_TiP_4_ a 1D conductivity mechanism in *c* direction, as usual via common faces of octahedral and tetrahedral voids, but avoiding the empty octahedral voids, is favoured over a 3D conductivity (Figure 13). The finding that initially unoccupied voids do not participate in the diffusion mechanism we have seen formerly from calculations using the maximum entropy method (MEM) on neutron diffraction data of the related lithium phosphidotetrelates and ‐aluminates [6, 9, 53]. For the second, apparently unoccupied void in Li_8_TiP_4_, that is 1/8 of the tetrahedral void (2*a* site) which is part of the favoured conduction path, a low Li content was detected during the refinement of the single crystal data of the Ta containing derivative as mentioned above. This indicates that occupation of this position might also be favoured in the pristine Li_8_TiP_4_, although the small amount of Li in this position could not be confirmed by powder data refinement. Remarkably, the lithium atom at the 4*d* site within the octahedral void is displaced from the center towards the vacant 2*a* site located in the face‐sharing tetrahedron, adopting a 3+3 Li coordination. The close proximity to the 2*a* site is consistent with a tendency for Li^+^ to diffuse towards this void, providing direct support for the pathway found by BVSE. Due to the unusually short distance between the 2*a* site and the Li4 atom at the 4*d* site, these two crystallographic positions cannot be occupied simultaneously. Thus, one Li atom at the 2*a* site will block the occupation of both neighboured 4*d* sites. This overcrowding of the participating atom sites as well as the low dimensionality of the conduction path may provide an explanation for the comparably low ionic conductivity. To access the Li1 site the highest energy barriers must be overcome (see Supporting Information) which confirms the finding of an extra signal in the ^6^Li NMR spectra for this atom.

The same calculations were carried out for the quaternary Ta containing compound based on the single crystal data as input (see Supporting information). The results are very close to those of Li_8_TiP_4_, the partial occupation of the empty tetrahedral void by Li does lead to marginally lower migration energy barriers.

## 4 Conclusion

The expansion of the compositional and structural variety of lithium phosphidotetrelate ion conductors by the inclusion of transition metal (*TM*) phosphides enables the discovery of numerous new compounds that preserve the characteristic motif of discrete tetrahedral *Tt*P_4_ or *TM*P_4_ structure units. Remarkably, the packing of these tetrahedra and the Li atoms gives rise to a wide range of structural configurations. Recently we reported on the synthesis and characterization of Li_7_TaP_4_ and Li_9.5_Ta_0.5_P_4_, both comprising discrete TaP_4_ tetrahedra. Notably, aliovalent substitution of Ta^5+^ by additional Li^+^ ions according to Li_7+5_ *_x_* Ta_1−_ *_x_* P_4_ led to a three‐orders‐of‐magnitude enhancement in lithium‐ion conductivity for *x* = 0.5 [55]. The two compounds crystalize in the cubic space groups *Pa* $\bar{3}$ and *Fm* $\bar{3}$ *m*, respectively, thus in contrast to the tetragonal symmetry of Li_8_TiP_4_ reported here, which crystallizes with space group *P*4_2_ *mc*. All structures can be traced back to a distorted variant of a 2×2×2 supercell of the antifluorite‐type Li_2_O type with phosphorus atoms occupying the oxygen positions, Ti and Ta atoms located on tetrahedral sites. Lithium atoms fill both, remaining tetrahedral and octahedral voids. A comparison of the void occupancies in Li_7_TaP_4_ and Li_9.5_Ta_0.5_P_4_ reveals that in in both compounds the tetrahedral voids are energetically preferred and are fully occupied. In contrast, the octahedral voids remain completely empty in Li_7_TaP_4_, whereas they exhibit a ≈ 50 % occupancy in Li_9.5_Ta_0.5_P_4_, reflecting the higher lithium content in this composition. These changes in lithium ion density and occupation of the octahedral voids lead to an increase of the ionic conductivity by three orders of magnitude from Li_7_TaP_4_ to Li_9.5_Ta_0.5_P_4_ [55]. Thus, partial occupation of the octahedral voids is generally a good indicator for Li ion conductivity. However, even though 50 % of the octahedral voids are occupied, the ionic conductivity of Li_8_TiP_4_ is similarly low as that of Li_7_TaP_4_. This is now fully explained by the crystal symmetry: In Li_8_TiP_4_, two symmetrically distinct octahedral voids are present: one is fully occupied, while the other remains vacant resulting in an average occupancy of 50 %. However, neither of the fully occupied nor completely empty octahedral sites appear to contribute to the ionic conduction mechanism.

In summary, the ternary lithium phosphidotitanate Li_8_TiP_4_ fits nicely in the series of ternary lithium phosphides. The difference in void occupancy and lithium ion density leads to ionic conductivities that differ by three orders of magnitude.

## Conflicts of Interest

The authors declare no Conflicts of Interest.

## Supporting information




**Supporting file1**: chem70705‐sup‐0001‐SuppMat.pdf

**Supporting file2**: chem70705‐sup‐0002‐Data.zip

## Data Availability

The data that support the findings of this study are available in the supplementary material of this article.
