# Supplementary material for: Structural Aspects of Lithium‐Ion Conduction in the Phosphidotitanate Li8TiP4 and Its Comparison With Li7+5 x Ta1− x P4 and Li8− x Ti1− x Ta x P4
Source: Chemistry. 2026 Jan 22;32(13):e03124. doi: 10.1002/chem.202503124 (PMC13047413; doi:10.1002/chem.202503124)
Supplement: Supplementary file 1 — Supporting file1: chem70705‐sup‐0001‐SuppMat.pdf [file CHEM-32-e03124-s001.pdf]

Structural Aspects of Lithium-Ion Conduction in the Phosphidotitanate Li_8TiP_4 and Its Comparison with $Li_{7+5x}Ta_{1-x}P_4$ and $Li_{8-x}Ti_{1-x}Ta_xP_4$

David Müller,[a] Tobias Kutsch,[b,e] Sabine Zeitz,[a] Viktor Hlukhyy,[a] Gabriele Raudaschl-Sieber,[c] Wilhelm Klein,[a,d] Thomas F. Fässler[a]*

[a] Technical University of Munich, TUM School of Natural Sciences, Chair of Inorganic Chemistry with Focus on New Materials, Lichtenbergstraße 4, 85748 Garching, Germany

[b] Technical University of Munich, TUM School of Natural Sciences, Chair of Technical Electrochemistry, Lichtenbergstraße 4, 85748 Garching, Germany

[c] Technical University of Munich, TUM School of Natural Sciences, Chair of Inorganic and Metal-Organic Chemistry, Lichtenbergstraße 4, 85748 Garching, Germany

[d] Technical University of Munich, TUM Catalysis Research Center, Ernst-Otto-Fischer-Straße 1, 85748 Garching, Germany

[e] TUMint·Energy Research GmbH, Lichtenbergstraße 4, 85748 Garching, Germany

Supporting Information

Content

Synthesis of Li_8TiP_4 (PXRD plots)	S-2
Single crystal structure determination of $Li_{8-x}Ti_{1-x}Ta_xP_4$ (x = 0.06)	S-3
Comparison of interatomic distances and angles in Li_8TiP_4 and $Li_{7.9367(6)}Ti_{0.9367(6)}Ta_{0.0633(6)}P_4$	S-6
Symmetry degradation according to Bärnighausen for Li_8TiP_4	S-7
Rietveld refinement results of Li_8TiP_4 in alternative settings	S-8
Calculated Raman modes	S-10
Overlap population	S-11
BVSE calculations for $Li_{8-x}Ti_{1-x}Ta_xP_4$ (x = 0.06)	S-12
Bode plot	S-14

Synthesis of Li_8TiP_4

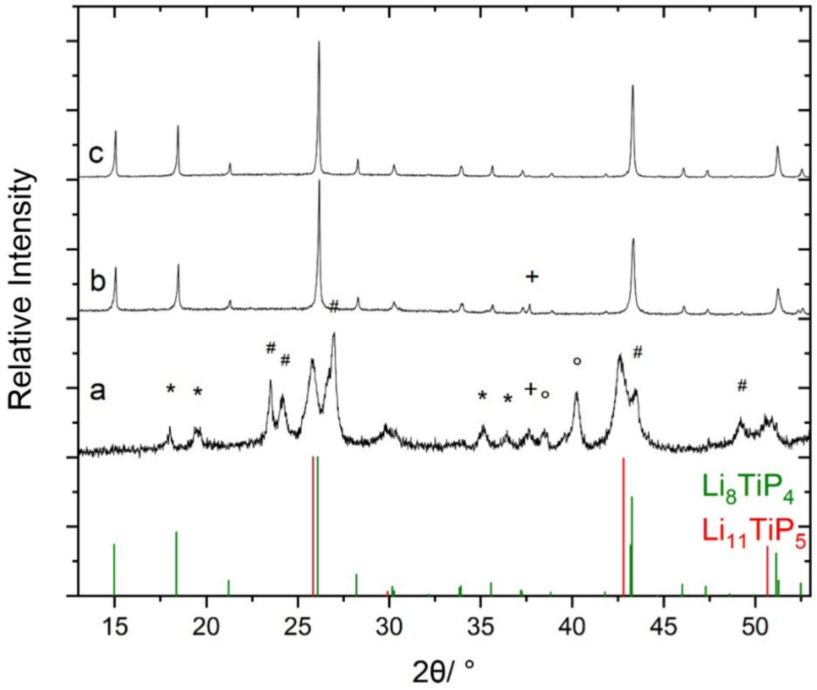

Figure S1: X-ray powder diffractograms (Cu $K_{\alpha1}$ radiation, room temperature) of a reactive mixture for synthesis of Li_8TiP_4: a) after ball milling; b) after annealing at 973 K for 24 h and slow cooling with 1 K/min; c) after annealing at 973 K for 24 h and subsequent quenching. After ball milling the disordered phase (red peaks, named as "$Li_{11}TiP_5$" according to the composition as assigned in [1]) is observed, as side phases LiP(*), Li_3P (#), TiP (+), and Ti (°) are detected. The ordered compound forms during the annealing step, shown here as green peaks.

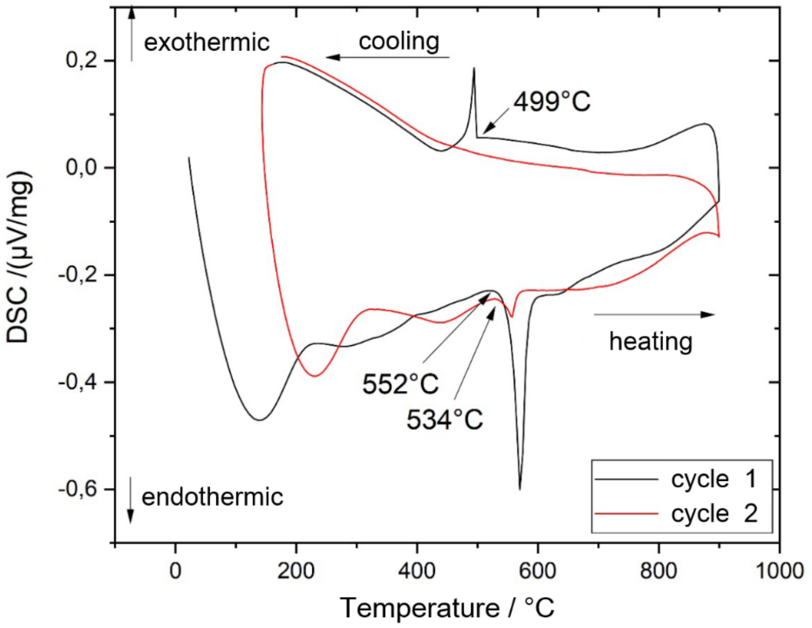

Figure S2: DSC measurement of the reactive mixture from a ball mill synthesis of Li_8TiP_4 with a heating and cooling rate of 10 K/min. The signals around 500°C have been attributed to the melting point of LiP, which is present in the first heating/cooling cycle but reacts to the ternary phase. Consequently, the signals disappear after the second heating period.

Crystal structure of $Li_{8-x}Ti_{1-x}Ta_xP_4$ (x = 0.06)

Data have been recorded for the same single crystal at room temperature, at 253 K, and at 150 K in order to check for changes of the Li atom ordering between the different tetrahedral and octahedral voids, or even for possible phase transitions to the related α- and β-phases. In the considered temperature range no significant changes have been observed. Tables S1 to S9 show the results of the structure determinations at the different temperatures.

Table S1: Crystallographic data and details of the single crystal structure determinations of $Li_{8-x}Ti_{1-x}Ta_xP_4$ at room temperature, at 253 K and at 150 K

refined composition	$Li_{7.9363(6)}Ti_{0.9363(6)}Ta_{0.0637(6)}P_4$	$Li_{7.9374(6)}Ti_{0.9374(6)}Ta_{0.0626(6)}P_4$	$Li_{7.9364(6)}Ti_{0.9364(6)}Ta_{0.0636(6)}P_4$
formula weight	235.33 g·mol^{-1}	235.19 g·mol^{-1}	235.32 g·mol^{-1}
temperature	293 K	253 K	150 K
crystal system		tetragonal	
space group		$P4_2mc$ (no. 105)	
a	8.3681(4) Å	8.3598(4) Å	8.3475(4) Å
c	5.9191(3) Å	5.9105(3) Å	5.8986(3) Å
V	414.49(4) Å^3	413.06(4) Å^3	411.02(4) Å^3
Z		2	
λ		0.71073 Å (Mo K_α)	
ρ_{calcd}	1.886 g·cm^{-3}	1.891 g·cm^{-3}	1.901 g·cm^{-3}
μ	2.476 mm^{-1}	2.471 mm^{-1}	2.496 mm^{-1}
2θ range	6.89° - 93.03°	6.89° - 92.96°	6.90° - 92.97°
collected refl.	11259	11183	11138
R_{int}	0.0190	0.0196	0.0181
independent refl.	1635	1627	1621
reflections > 2 $\sigma(I)$	1426	1421	1469
Flack parameter	0.46(3)	0.49(3)	0.47(3)
param. / restraints	46 / 6	46 / 6	43 / 0
R_1 [I > 2 $\sigma(I)$ / all data]	0.0170 / 0.0246	0.0174 / 0.0246	0.0162 / 0.0213
wR_2 [I > 2 $\sigma(I)$ / all data]	0.0260 / 0.0272	0.0280 / 0.0291	0.0269 / 0.0277
goodness of fit	1.016	0.989	1.026
max./min. diff. el. density	0.269 / −0.431 e/Å^{-3}	0.284 / −0.745 e/Å^{-3}	0.311 / −0.589 e/Å^{-3}
depository no.	CSD-2465968	CSD-2465969	CSD-2465970

Table S2. Atom positions, Wyckoff sites, site occupation factors and isotropic displacement parameters [Å^2] in $Li_{7.9363(6)}Ti_{0.9363(6)}Ta_{0.0637(6)}P_4$ at room temperature.

Atom	Wyckoff site	*x*	*y*	*z*	*s.o.f.*	U_{eq}
Li1	2*c*	0	½	0.740(2)	1	0.0145(7)
Li2	8*f*	0.2694(3)	0.2492(3)	0.2445(14)	1	0.0199(5)
Li3	2*b*	½	½	0.2862(8)	1	0.0220(9)
Li4	4*d*	0	0.1655(6)	0.0257(8)	0.9363(6)	0.0426(10)
Li5	2*a*	0	0	0.151(13)	0.0637(6)	0.023(16)
Ti	2*c*	0	½	¼	0.9363(6)	0.00655(3)
Ta	2*c*	0	½	¼	0.0637(6)	0.00655(3)
P1	4*e*	0.23273(6)	½	0.01916(18)	1	0.00907(9)
P2	4*d*	0	0.25836(7)	0.46997(18)	1	0.00939(9)

Table S3. Anisotropic displacement parameters [Å^2] of the single crystal structure determination of $Li_{7.9363(6)}Ti_{0.9363(6)}Ta_{0.0637(6)}P_4$ at room temperature.

Atom	U_{11}	U_{22}	U_{33}	U_{12}	U_{13}	U_{23}
Li1	0.013(3)	0.021(3)	0.010(2)	0	0	0
Li2	0.0193(10)	0.0204(11)	0.0200(9)	0.0027(9)	−0.0067(14)	0.0025(6)
Li3	0.028(2)	0.0185(18)	0.019(2)	0	0	0
Li4	0.0187(15)	0.047(2)	0.062(3)	−0.027(2)	0	0
Li5	0.015(19)	0.04(2)	0.02(2)	0	0	0
Ti	0.00691(16)	0.00587(16)	0.00686(5)	0	0	0
Ta	0.00691(16)	0.00587(16)	0.00686(5)	0	0	0
P1	0.00917(18)	0.00925(19)	0.00880(17)	0	0.00106(14)	0
P2	0.0094(2)	0.00791(16)	0.01086(19)	0.00103(14)	0	0

Table S4. Atom positions, Wyckoff sites, site occupation factors and isotropic displacement parameters [Å^2] in $Li_{7.9374(6)}Ti_{0.9374(6)}Ta_{0.0626(6)}P_4$ at 253 K.

Atom	Wyckoff site	*x*	*y*	*z*	*s.o.f.*	U_{eq}
Li1	2*c*	0	½	0.745(2)	1	0.0141(6)
Li2	8*f*	0.2698(3)	0.2497(3)	0.2414(13)	1	0.0164(5)
Li3	2*b*	½	½	0.2863(9)	1	0.0177(8)
Li4	4*d*	0	0.1657(6)	0.0256(8)	0.9374(6)	0.0375(10)
Li5	2*a*	0	0	0.159(14)	0.0626(6)	0.017(15)
Ti	2*c*	0	½	¼	0.9374(6)	0.00577(3)
Ta	2*c*	0	½	¼	0.0626(6)	0.00577(3)
P1	4*e*	0.23302(7)	½	0.01857(17)	1	0.00792(9)
P2	4*d*	0	0.25823(7)	0.46947(17)	1	0.00841(9)

Table S5. Anisotropic displacement parameters [Å^2] of the single crystal structure determination of $Li_{7.9374(6)}Ti_{0.9374(6)}Ta_{0.0626(6)}P_4$ at 253 K.

Atom	U_{11}	U_{22}	U_{33}	U_{12}	U_{13}	U_{23}
Li1	0.009(3)	0.023(3)	0.0112(15)	0	0	0
Li2	0.0162(10)	0.0117(10)	0.0160(12)	0.0019(9)	−0.0033(13)	0.0019(6)
Li3	0.0212(19)	0.0159(18)	0.016(2)	0	0	0
Li4	0.0152(14)	0.043(2)	0.054(3)	−0.023(2)	0	0
Li5	0.011(19)	0.03(2)	0.010(19)	0	0	0
Ti	0.00643(16)	0.00493(16)	0.00597(5)	0	0	0
Ta	0.00643(16)	0.00493(16)	0.00597(5)	0	0	0
P1	0.00802(18)	0.00816(19)	0.00757(16)	0	0.00053(14)	0
P2	0.0085(2)	0.00721(16)	0.00949(19)	0.00096(14)	0	0

Table S6. Atom positions, Wyckoff sites, site occupation factors and isotropic displacement parameters [Å^2] in $Li_{7.9364(6)}Ti_{0.9364(6)}Ta_{0.0636(6)}P_4$ at 150 K.

Atom	Wyckoff site	*x*	*y*	*z*	*s.o.f.*	U_{eq}
Li1	2*c*	0	½	0.740(2)	1	0.0100(6)
Li2	8*f*	0.2700(3)	0.2496(3)	0.2436(12)	1	0.0132(4)
Li3	2*b*	½	½	0.2840(6)	1	0.0139(8)
Li4	4*d*	0	0.1647(5)	0.0270(7)	0.9364(6)	0.0266(7)
Li5	2*a*	0	0	0.169(11)	0.0636(6)	0.00405(8)
Ti	2*c*	0	½	¼	0.9364(6)	0.00405(3)
Ta	2*c*	0	½	¼	0.0636(6)	0.00405(3)
P1	4*e*	0.23334(6)	½	0.01872(15)	1	0.00551(8)
P2	4*d*	0	0.25795(6)	0.46997(16)	1	0.00581(8)

Table S7. Anisotropic displacement parameters [Å^2] of the single crystal structure determination of $Li_{7.9364(6)}Ti_{0.9364(6)}Ta_{0.0636(6)}P_4$ at 150 K.

Atom	U_{11}	U_{22}	U_{33}	U_{12}	U_{13}	U_{23}
Li1	0.011(3)	0.012(3)	0.0070(19)	0	0	0
Li2	0.0117(8)	0.0146(9)	0.0133(8)	0.0012(8)	−0.0037(11)	0.0017(5)
Li3	0.0174(16)	0.0132(14)	0.0110(18)	0	0	0
Li4	0.0146(12)	0.0287(17)	0.0366(18)	−0.0129(14)	0	0
Li5	0.00415(14)	0.00374(14)	0.00428(5)	0	0	0
Ti	0.00415(14)	0.00374(14)	0.00428(5)	0	0	0
Ta	0.00415(14)	0.00374(14)	0.00428(5)	0	0	0
P1	0.00552(15)	0.00555(16)	0.00556(15)	0	0.00032(12)	0
P2	0.00575(17)	0.00515(14)	0.00660(16)	0.00058(12)	0	0

Table S8: Comparison of selected interatomic distances and angles in Li_8TiP_4 from Rietveld refinement and $Li_{8-x}Ti_{1-x}Ta_xP_4$ from single crystal structure determination at RT.

	**Li_8TiP_4 (RT)**	**$Li_{8-x}Ti_{1-x}Ta_xP_4$ (RT)**
Atoms	Distance [Å]	Distance [Å]
TM-P1 (2×)	2.384(6)	2.3790(8)
TM-P2 (2×)	2.394(6)	2.4050(7)
Li1-P1 (2×)	2.59(3)	2.556(9)
Li1-P2 (2×)	2.53(3)	2.576(8)
Li2-P1	2.493(18)	2.506(5)
Li2-P1	2.54(2)	2.527(5)
Li2-P2	2.626(18)	2.621(5)
Li2-P2	2.64(2)	2.646(5)
Li3-P1 (2×)	2.631(13)	2.627(3)
Li3-P1 (2×)	2.758(15)	2.739(3)
Li4-P2 (2×)	2.624(8)	2.589(3)
Li4-P2	2.78(2)	2.742(4)
Li4-P2	3.28(2)	3.380(5)
Li4-P1 (2×)	3.34(2)	3.410(4)
Li5-P2 (2×)	-	2.41(3)
Li5-P2 (2×)	-	2.87(5)
Li5-Li4 (2×)	-	1.57(4)
Li5-Li4 (2×)	-	2.62(7)
TM-Li1	2.79(4)	2.898(13)
TM-Li1	3.11(4)	3.022(13)
TM-Li2 (4×)	3.075(10)	3.0803(18)
TM-Li4 (2×)	3.071(14)	3.098(4)
P1-P1	3.89(1)	3.8951(11)
P1-P2 (4×)	3.87(1)	3.8733(3)
P2-P2^{i}	4.05(1)	4.0442(11)
Atoms	Angle [°]	Angle [°]
P1-*TM*-P1	108.6(1)	109.90(4)
P1-*TM*-P2 (4×)	108.1(1)	108.116(5)
P2-*TM*-P2	115.7(1)	114.44(5)
P1-Li1-P1	96.7(1)	99.2(5)
P1-Li1-P2 (4×)	113.5(1)	113.646(13)
P2-Li1-P2	106.3(1)	103.5(5)

Figure S4. Symmetry degradation according to Bärnighausen for Li_8TiP_4 starting from Li_3Bi. At the final stage the positional parameters of $Li_{8-x}Ti_{1-x}Ta_xP_4$ were applied instead of those of pure Li_8TiP_4, owing to the presence of the additional Li5 atom.

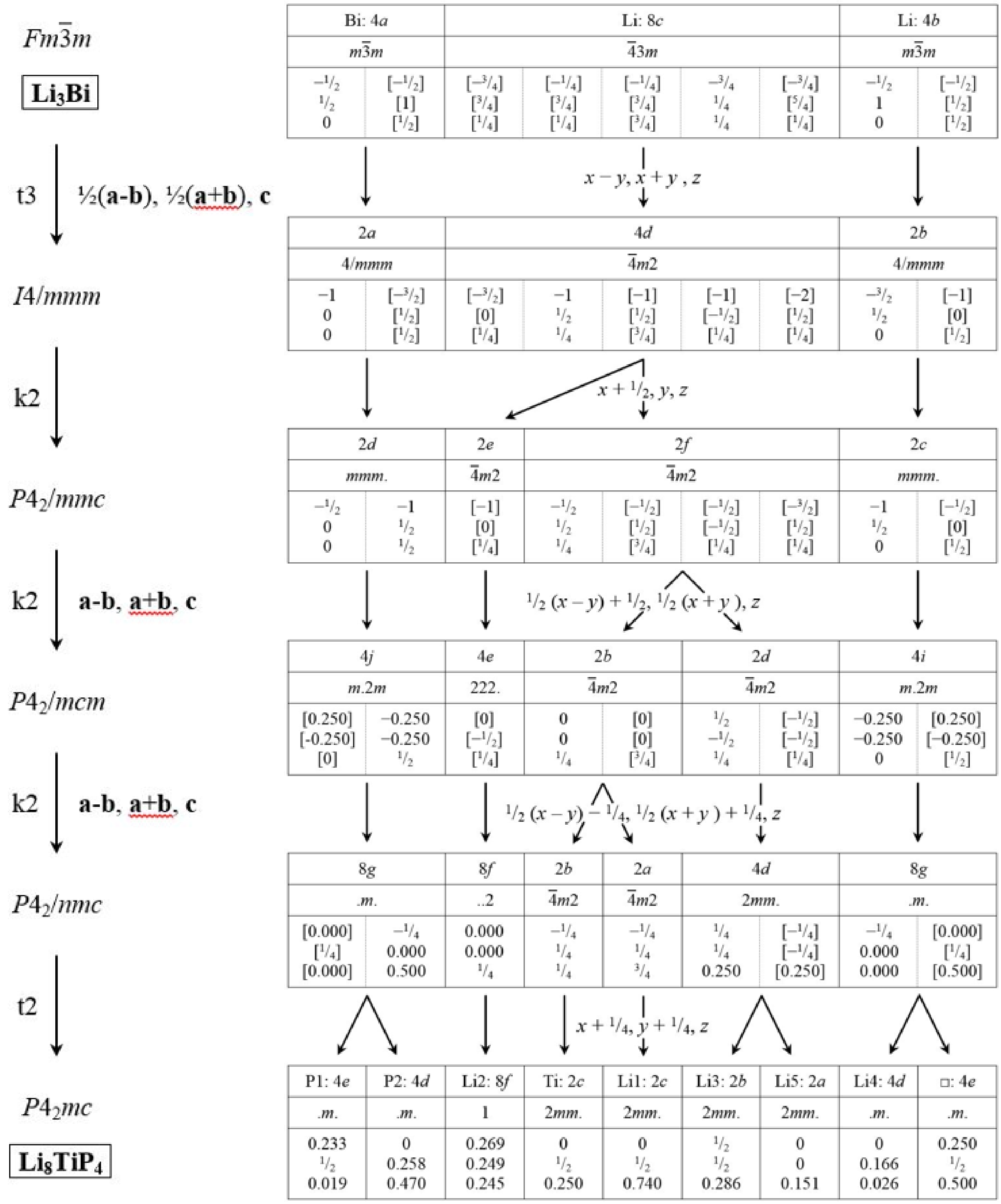

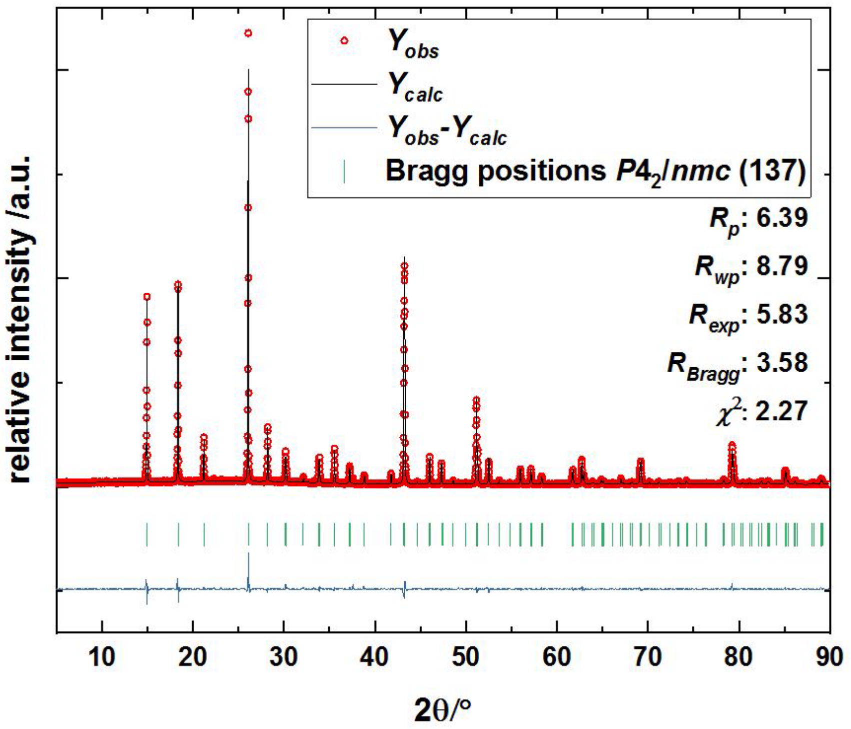

Figure S5: Result from Rietveld refinement of Li_8TiP_4 in space group *P*4$_2$/*nmc* (no. 137), measured with Cu $K_{\alpha 1}$ radiation. The measured reflections are represented by red circles, the fit curve is shown as black line, green stripes mark the Bragg positions and the blue line gives the difference between measured reflections and the fit curve.

Table S9: Results of the Rietveld refinements of Li_8TiP_4 at room temperature in space group *P*4$_2$/*nmc* (no. 137) with fixed occupations and in *P*4$_2$*mc* (no. 105) with free occupations for Li atoms

composition	Li_8P_4Ti	Li_8P_4Ti
space group	*P*4$_2$/*nmc* (no. 137)	*P*4$_2$*mc* (no. 105)
formula weight	227.30 g mol^{-1}	225.25 g mol^{-1}
temperature	293 K	293 K
unit cell	*a* = 8.37177(6) Å	*a* = 8.37543(3) Å
	c = 5.90109(5) Å	*c* = 5.90459(2) Å
V	413.587(7) Å^3	414.194(2) Å^3
Z	2	
ρ_{calc}	1.825 g cm^{-3}	1.806 g cm^{-3}
λ	Cu $K_{\alpha 1}$ (1.540598 Å)	
μ	15.216	15.193
2ϑ range	5° - 90°	
R_p	6.39	3.32
R_{wp}	8.79	4.74
R_{exp}	5.83	3.07
χ^2	2.27	2.38
R_{Bragg}	3.58	2.03
R_f	3.04	2.25

Table S10: Atom positions, Wyckoff sites, isotropic displacement parameters [Å^2], and site occupation factors of Li_8TiP_4 from Rietveld refinement in space group *P*4$_2$/*nmc* (no. 137).

Atom	Wyckoff pos.	*x*	*y*	*z*	U_{iso}	s.o.f.
Ti1	2*b*	0.25	0.75	0.75	0.0064(6)	1
P1	8*g*	0.01297	0.75	-0.0252(2)	0.0051(4)	1
Li1	8*f*	-0.0099(7)	0.4901(7)	0.75	0.020(2)	1
Li2	2*a*	0.25	0.75	0.25	0.020(2)	1
Li3	4*d*	0.75	0.75	0.212	0.020(2)	1
Li4	8*g*	0.75	0.588	0.017	0.020(2)	0.125

Table S11: Atom positions, Wyckoff sites, isotropic displacement parameters [Å^2], and site occupation factors of Li_8TiP_4 from Rietveld refinement in space group *P*4$_2$*mc* (no. 105) with free occupational parameters for all Li atoms.

Atom	Wyckoff pos.	*x*	*y*	*z*	U_{iso}	s.o.f.
Ti1	2*c*	0	1/2	0.25	0.0072(5)	1
P1	4*e*	0.2308(4)	1/2	0.0145(16)	0.0082(14)	1
P2	4*d*	0	0.2577(4)	0.4656(17)	0.0068(13)	1
Li1	2*c*	0	1/2	0.728(8)	0.006(6)	0.97(2)
Li2	8*f*	0.2679(14)	0.2491(14)	0.238(5)	0.006(3)	0.976(13)
Li3	2*b*	1/2	1/2	0.283(4)	0.018(9)	0.97(3)
Li4	4*d*	0	0.1745(17)	0.008(3)	0.042(8)	0.93(3)

Table S12: Raman modes of Li_8TiP_4, comparison of measured and calculated modes.

DFT	corrected	Symmetry	Measurement	DFT	corrected	Symmetry	Measurement
104.76	98.47	E		318.94	299.80	E	
104.76	98.47	E		328.10	308.41	E	
124.77	117.28	B1	120	328.10	308.41	E	
138.09	129.80	E		329.99	310.19	B1	
138.09	129.80	E		337.29	317.05	A1	
163.66	153.84	B2		337.89	317.62	E	
168.44	158.33	E		337.89	317.62	E	
168.44	158.33	E		351.47	330.38	B1	
177.39	166.75	A1	160	356.27	334.89	A1	
186.95	175.73	A1	176	358.21	336.72	E	
193.65	182.03	E		358.21	336.72	E	
193.65	182.03	E		367.79	345.72	B1	
203.22	191.03	E	191	368.51	346.40	A1	345
203.22	191.03	E		384.01	360.97	A1	
216.59	203.59	E		384.01	360.97	A1	
216.59	203.59	E		390.88	367.43	B1	
221.26	207.98	E	212	403.25	379.06	A1	381
222.17	208.84	B1		403.55	379.34	A1	
231.37	217.49	E		407	382.58	E	
231.37	217.49	E		407	382.58	E	
233.46	219.45	B2		411.31	386.63	B2	
235.1	220.99	A1	224	416.32	391.34	E	389
238.23	223.94	B1		416.32	391.34	E	
240.08	225.68	E		441.61	415.11	E	415
240.08	225.68	E		444.24	417.59	B2	
261.47	245.78	A1		444.58	417.91	E	
264.34	248.48	B2	247	444.58	417.91	E	
269.71	253.53	E		464.97	437.07	B1	
269.71	253.53	E		465.2	437.29	E	
297.78	279.91	E		465.2	437.29	E	440
297.78	279.91	E		485.98	456.82	A1	
306.33	287.95	B2	279	497.49	467.64	E	470
312.82	294.05	B1		497.49	467.64	E	
318.94	299.80	E	300	506.64	476.24	B1	479

Table S13: Atomic distances [Å] and overlap population for all nearest neighbors in Li_8TiP_4.

Atom A	Atom B	r_{AB}	overlap	Atom A	Atom B	r_{AB}	overlap
Ti1	P2	2.369	0.24	P2	Li2	2.479	0.085
P1		2.383	0.245	Li2		2.523	0.08
Li1		2.896	0.024	Li1		2.529	0.084
Li1		2.979	0.017	Li4		2.608	0.076
Li2		3.094	0.015	Li4		2.743	0.066
Li3		3.095	0.018	Li1	Li2	2.817	0.009
P1	Li3	2.585	0.082	Li3		3.233	0.005
Li1		2.592	0.081	Li2	Li3	2.695	0.01
Li2		2.623	0.074	Li3		2.812	0.01
Li2		2.644	0.072	Li3	Li3	2.773	0.007
Li3		2.723	0.063	Li4	Li4	2.938	0.007

Bond Valence Site Energy (BVSE) Calculations for $Li_{8-x}Ti_{1-x}Ta_xP_4$ (x = 0.06)

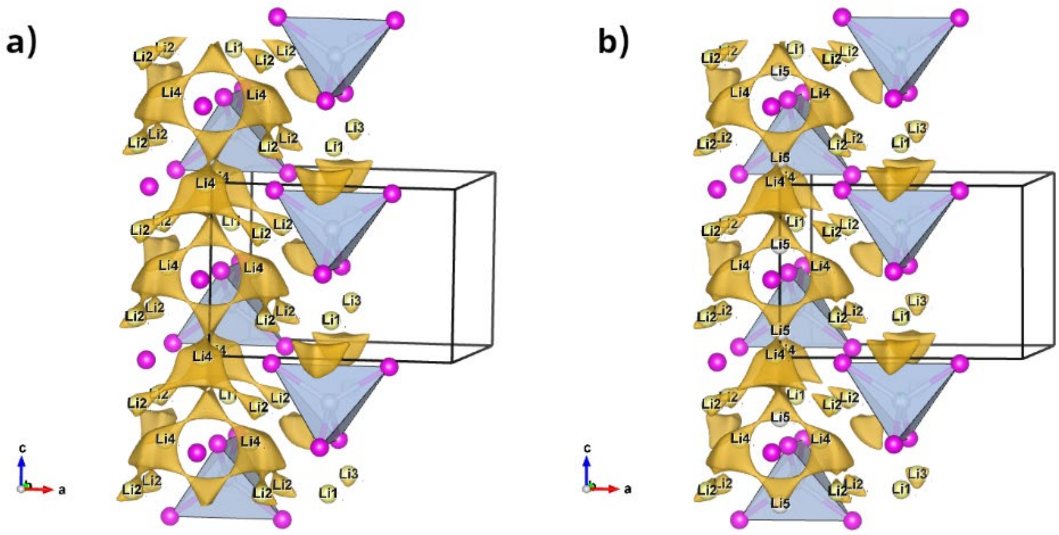

Figure S6: Comparison of the migration pathways through the structures of Li_8TiP_4 (a) and $Li_{7.9363(6)}Ti_{0.9363(6)}Ta_{0.0637(6)}P_4$ at room temperature (b). The free tetrahedral void in Li_8TiP_4 is partially occupied (Li5) in the Ta-doped compound.

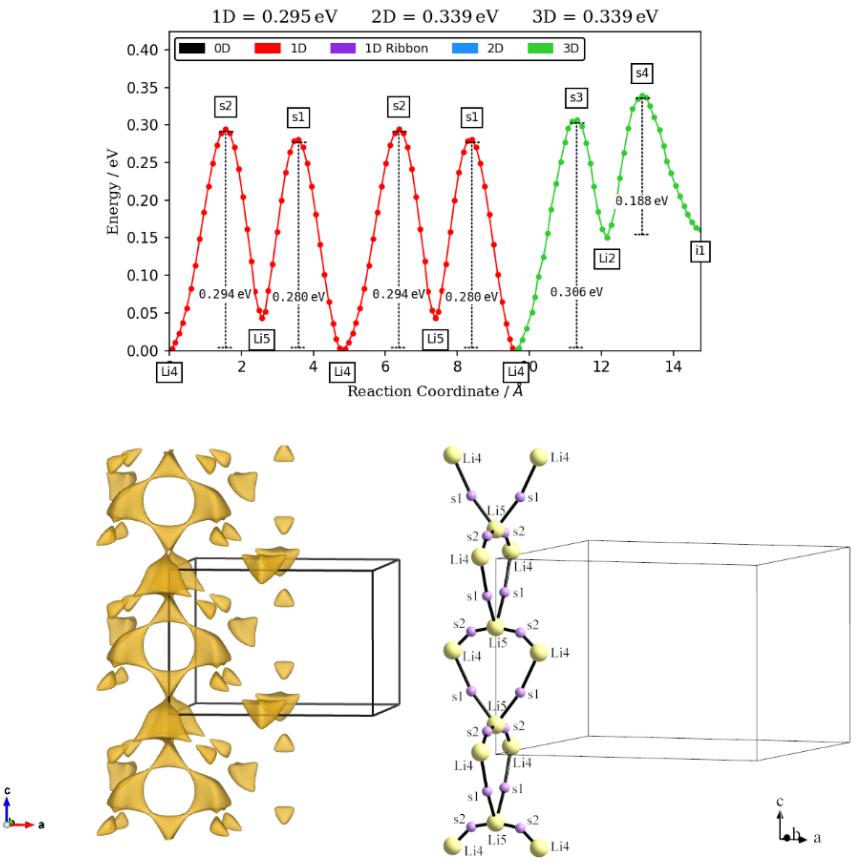

Figure S7: Lowest migration barriers for $Li_{7.9363(6)}Ti_{0.9363(6)}Ta_{0.0637(6)}P_4$ at room temperature and the resulting isosurface and 1D pathway along the *c* axis. The values are close to those of pure Li_8TiP_4, with slightly higher relative energies for Li2 and the free octahedral void.

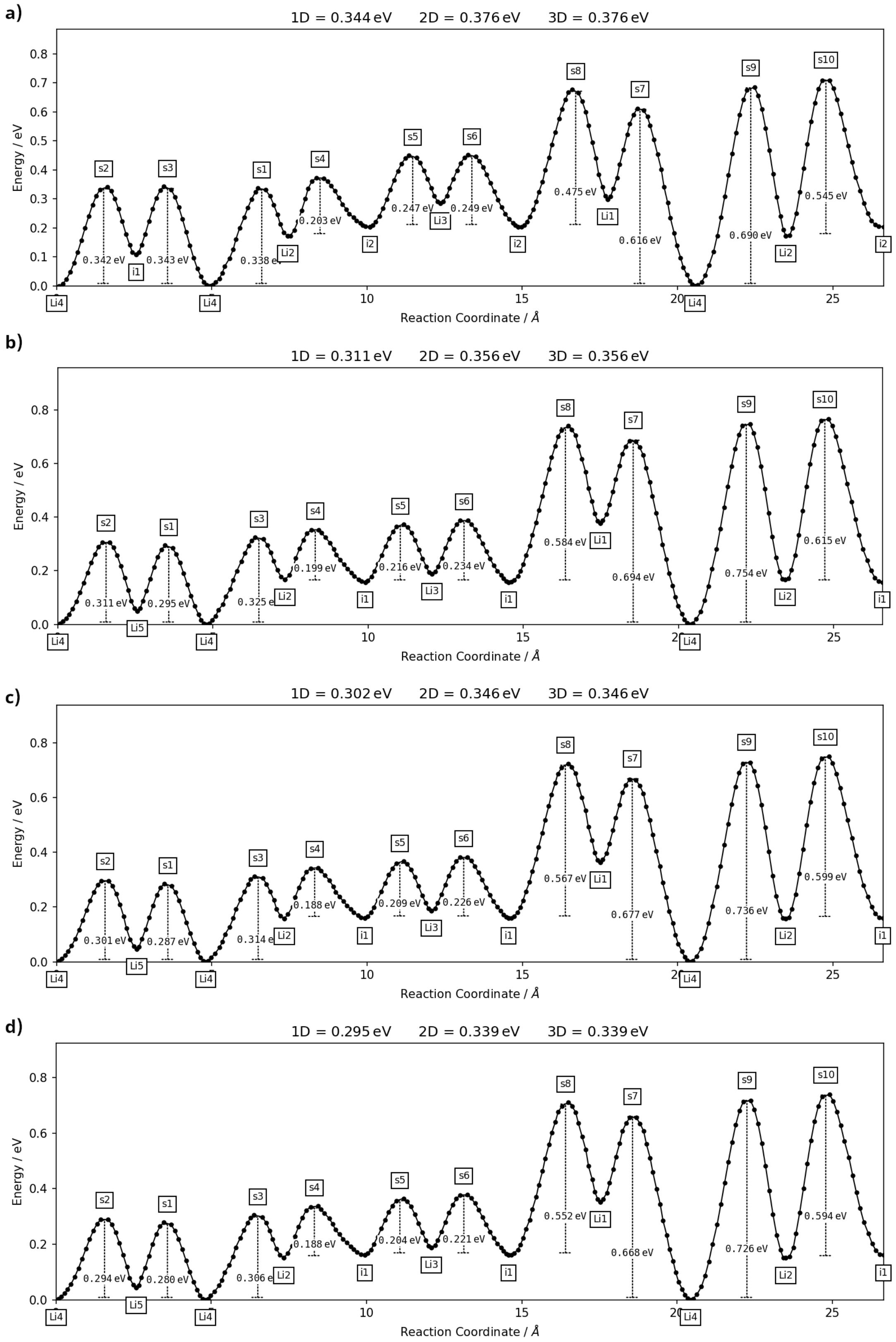

Figure S8: Migration barriers including all crystallographic Li sites for Li_8TiP_4 (a) and for $Li_{7.9363(6)}Ti_{0.9363(6)}Ta_{0.0637(6)}P_4$ at 150 K (b), 253 K (c), and room temperature (d). The unoccupied octahedral void is assigned as i2 in a) and as i1 in b) - d). The energy values are of the Ta-doped compound decrease with increasing temperature and are slightly lower than those for pure Li_8TiP_4.

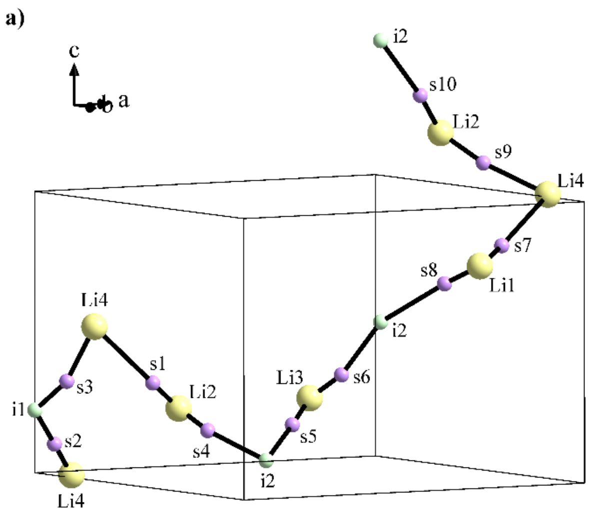

Figure S9: Migration pathways including all crystallographic Li sites as a continuous 3D pathway according to the barriers shown in Fig. S15 for Li_8TiP_4 (a) and for $Li_{7.9363(6)}Ti_{0.9363(6)}Ta_{0.0637(6)}P_4$ at room temperature (b). The unoccupied octahedral void is assigned as "i2" in a) and as "i1" in b).

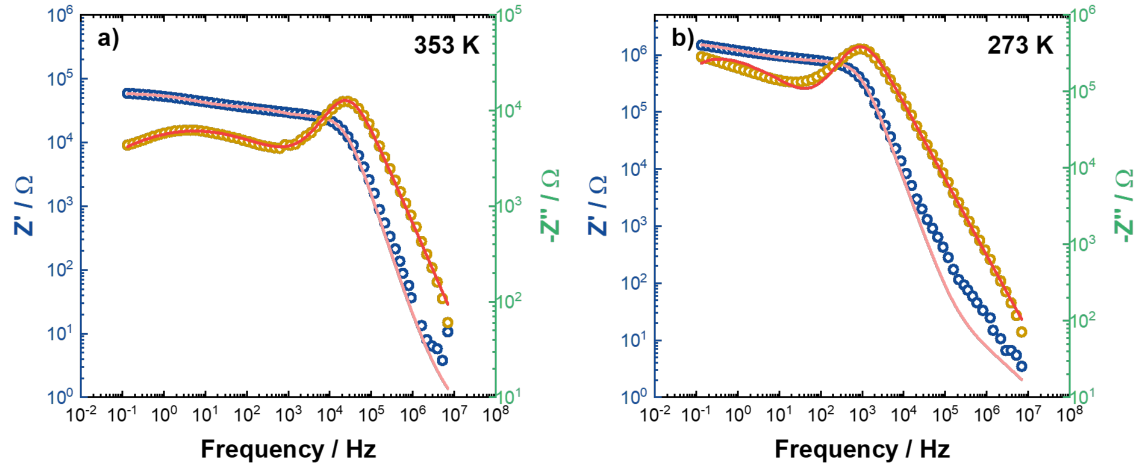

Figure S10: Bode plot of the impedances collected at 353 K (a) and 273 K (b) as well as their corresponding fitting (red lines) using the equivalent circuit model shown in Fig. 12.c.

References

[1] A. Adam, H.-U. Schuster, *Z. Anorg. Allg. Chem.* **1991**, *597*, 33-39.
